# The causal effect of iron status on risk of anxiety disorders: A two-sample Mendelian randomization study

**DOI:** 10.1371/journal.pone.0300143

**Published:** 2024-03-28

**Authors:** Ruiying Yin, Qi Gao, Guangzhen Fu, Qiang Zhao

**Affiliations:** Department of Clinical Laboratory, The First Affiliated Hospital of Zhengzhou University, Key Clinical Laboratory of Henan Province, Zhengzhou, Henan, China; The First Hospital of Jilin University, CHINA

## Abstract

**Objectives:**

Observational studies had investigated the association of iron metabolism with anxiety disorders. The conclusions were inconsistent and not available to reveal the causal or reverse-causal association due to the confounding. In this study we estimated the potential causal effect of iron homeostasis markers on anxiety disorders using two-sample Mendelian randomization (MR) analysis.

**Methods:**

Summary data of single nucleotide polymorphisms (SNPs) associated with four iron-related biomarkers were extracted from a recent report about analysis of three genome-wide association study (GWAS), the sample size of which ranged from 131471 to 246139 individuals. The corresponding data for anxiety disorders were from Finngen database (20992 cases and 197800 controls). The analyses were mainly based on inverse variance weighted (IVW) method. In addition, the heterogeneity and pleiotropy of the results were assessed by Cochran’s Q test and MR-Egger regression.

**Results:**

Basing on IVW method, genetically predicted serum iron level, ferritin and transferrin had negative effects on anxiety disorders. The odd ratios (OR) of anxiety disorders per 1 standard deviation (SD) unit increment in iron status biomarkers were 0.922 (95% confidence interval (CI) 0.862–0.986; p = 0.018) for serum iron level, 0.873 (95% CI 0.790–0.964; p = 0.008) for log-transformed ferritin and 0.917 (95% CI 0.867–0.969; p = 0.002) for transferrin saturation. But no statical significance was found in the association of 1 SD unit increased total iron-binding capacity (TIBC) with anxiety disorders (OR 1.080; 95% CI 0.988–1.180; p = 0.091). The analyses were supported by pleiotropy test which suggested no pleiotropic bias.

**Conclusion:**

Our results indicated that genetically determined iron status biomarkers causally linked to the risk of anxiety disorders, providing valuable insights into the genetic research and clinical intervention of anxiety disorders.

## Introduction

In recent decades, anxiety disorders become prevalent and the global burden that caused by the diseases make it an important health issue about psychiatry. The chronicity and comorbidity of anxiety disorders lead to huge consequences of health lost. Because of that, the World Health Organization (WHO) listed anxiety disorders as one of the top contributors of global disability [1, 2]. Exploration of the factors involving in the processes of anxiety disorders will make a difference for the prevention and treatment. However, anxiety disorders usually manifest as complex physical and mental conditions which are susceptible to multiple environmental factors. Essential elements including iron, zinc, copper, selenium, and manganese have been suggested to be associated with mental disorders. A cross-sectional analysis of National Health and Nutrition Examination Survey (NHANES) 2011–2016 revealed that higher serum zinc, ratio of zinc to copper and zinc to selenium were associated with a lower risk of self-reported sleep disorders in US adults [3]. Circulating selenium level was also reported to negatively associated with the risk of schizophrenia [4]. In the pathophysiology of anxiety, these essential elements were demonstrated to play an important role and may be a prognostic marker and a tool for clinical intervention [5, 6]. As to iron, systemic iron status is usually assessed by several biomarkers in clinical practice: serum iron, ferritin, transferrin saturation, transferrin, and total iron-binding capacity (TIBC) [7]. Beside serum iron, ferritin and transferrin saturation represent iron status positively, while transferrin and TIBC do inversely [8]. A comparative observational study showed that in patients with anxiety disorders, serum iron showed significantly higher level [6]. However, some researchers demonstrated different conclusions. According to a cross-sectional study, the correlation between serum ferritin level and anxiety was not found [9]. A meta-analysis suggested element metals including iron were not superior on alleviating perinatal anxiety and depression, when comparing with placebo [10]. Thus, whether iron status affects anxiety disorders remains to be controversial.

These inconsistent findings mentioned above may be due to the inherent defects during conducting observational studies. For example, it is usually difficult to avoid residual confounding and reverse causality thoroughly. Therefore, the Mendelian randomization (MR) study is developed as an innovative approach to assess the causal effects of exposure(s) on outcome(s). In MR analyses, genetic variants like single-nucleotide polymorphisms (SNPs) that strongly correlated with exposure are extracted as instrumental variables (IVs) [11]. Because genetic variants are randomly assigned during meiosis and unchangeable after that, the difference of SNPs among individuals precedes the occurrence of any disease naturally. Therefore, in MR studies the interferences of confounding variables and possible reverse causality can be minimized effectively [12]. In particular, MR study depends on public genome-wide association study (GWAS) summary data with large sample, which avoids ethical issues and reduces error during the analyses. Hence, in present study we conducted MR analyses to examine whether genetically predicted iron status biomarkers affect the risk of anxiety disorders. The causal associations of serum iron level, ferritin, transferrin saturation and TIBC with anxiety disorders were estimated, which may provide more evidence and proper suggestions to the intervention of anxiety disorders.

## Materials and methods

### Study design

**Fig 1:**
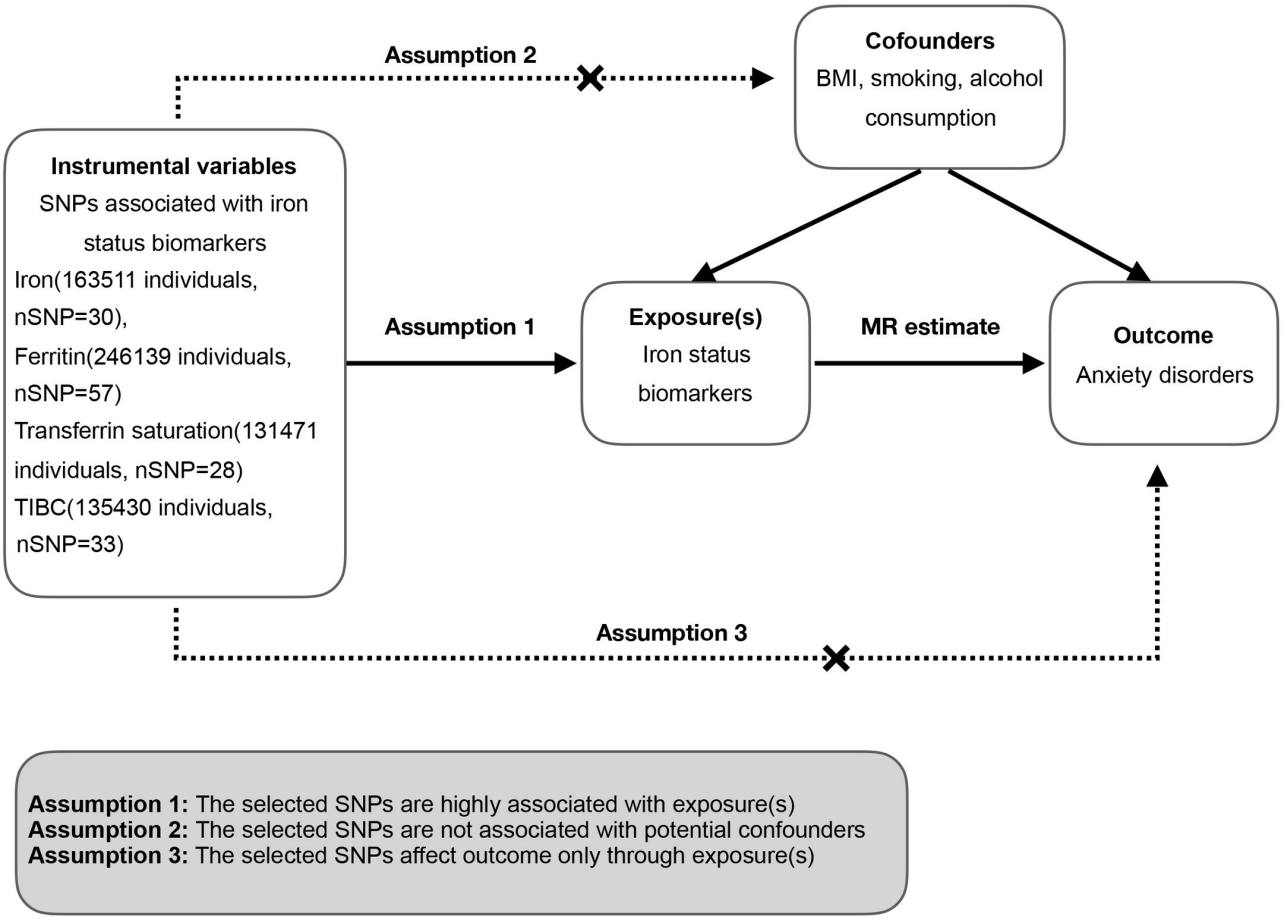
Overview of the two-sample MR study design. SNP, single nucleotide polymorphism; TIBC, total iron-binding capacity; BMI, body mass index.

We performed a two-sample MR study using summary-level data from different GWAS for the iron status as exposures and anxiety disorders as outcome respectively. Iron status was represented by four biomarkers comprehensively: serum iron, ferritin, transferrin saturation and TIBC. Dataset related to each iron biomarker was subjected to two-sample MR analysis as an exposure respectively. The overall study design is shown in Fig 1. It was set to conform to three key assumptions: 1) The selected IVs (refer to SNPs in our study) are highly associated with exposure(s); 2) The selected SNPs are not associated with potential confounders; 3) The selected SNPs affect outcome only through exposure(s).

Ethical approval and participant consent were received by local ethics committees according to the original publications or public database.

### Data sources for iron status biomarkers

Datasets related to iron status biomarkers was derived from the study published in 2021 by Bell S et al. [7], which analyzed three GWAS data from Iceland (deCODE genetics, 1990–2017), the UK (INTERVAL study, 2012–2014) and Denmark (2010–2019, Danish Blood Donor Study). Summary-level data were downloaded from http://www.decode.com/summarydata/ as directed in the study. The four iron homeostasis biomarkers included serum iron (SD = 7.76μmol/L, N = 163511), ferritin (log-transformed, SD = 1.08μg/L, N = 246139), transferrin saturation (SD = 13.25%, N = 131471) and TIBC (SD = 14.14μmol/L, N = 135430). As the researchers reported, each biomarker was transformed to a standard normal distribution through rank-based inverse normal transformation (separately for each sex). A generalized additive model was used to adjust for age. For UK cohort the biomarkers were adjusted for menopausal status, ABO blood group, BMI, smoking levels, alcohol levels and iron supplementation status.

### GWAS data of anxiety disorders

GWAS summary statistics for anxiety disorders was extracted from the Finngen database (https://r5.finngen.fi/, ID: KRA_PSY_ANXIETY, Year: 2021). The data were obtained from 218792 individuals including 20992 cases and 197800 controls with European ancestry. Anxiety disorders were defined by ICD8/9/10 based on hospital admission records.

### Selection of genetic IVs for iron status biomarkers

According to the three basic assumptions, a stepwise screening of SNPs as IVs for each exposure was performed using R software. Firstly, SNPs strongly associated with each biomarker (p<5×10^−8^) were extracted from the summary-level data of iron status biomarkers mentioned above. Secondly, independent SNPs were identified by a linkage-disequilibrium threshold of r^2^< 0.001 within a 10000 kb window, using a clumping procedure in R software. rs1800562, rs1799945 and rs855791 were not excluded because they were missense mutation of HFE and TMPRSS6 genes. HFE and TMPRSS6 are important proteins that directly participant in the signaling pathways of iron status regulation [7]. Next, we searched the selected SNPs in Phenoscanner database (http://www.phenoscanner.medschl.cam.ac.uk/) to identify whether they were associated with potential confounders including smoking [13], alcohol intake [14], body mass index [15] and outcome (i.e., anxiety disorders). SNPs highly associated with these phenotypes (p<5×10^−8^) were excluded. During extracting exposures associated SNPs from the outcome, proxy SNPs were identified in cases where the IVs cannot be matched in the outcome dataset. If no proxy SNPs were available, the SNPs will be dropped. When harmonizing the IVs and outcome data, we removed palindromic SNPs. Finally, F statistic parameters calculated with the explained variance (R^2^) and sample size (N) were used to determine the SNPs as powerful enough IVs or not [16]. F>10 is usually suggested as the threshold for MR analysis. All the SNPs selected in the present study showed high F value (F>10, S1 Table).

### MR estimates

Before final MR analysis, outliers were screened through the MR PRESSO test in R project with 5000 bootstrap replications and then excluded if the results suggested outliers existed. Inverse variance weighted (IVW) analysis as the main approach was used to estimate the effect of iron biomarkers on anxiety disorders. We employed the random effects model in case of heterogeneity and fixed effects model when no heterogeneity was found. Odd ratios (OR) were used in the present the MR estimates, which are scaled to one standard deviation (SD) increment of genetically predicted each iron status biomarker. MR Egger, Weighted median, Simple mode and Weighted mode were also conducted as additional analyses with different assumptions. Power of IVW analysis were evaluated (https://sb452.shinyapps.io/power/). Heterogeneity across the selected SNPs as IVs was assessed by Cochran’s Q test. Intercepts of MR-Egger regression was employed to evaluate the horizontal pleiotropy and bias caused by ineffective IVs. A “leave-one-out” sensitivity analysis was implemented to determine whether the causal association between exposure and outcome were any driven by any single SNPs.

All analyses were performed using R project (version 4.2.2, https://www.r-project.org/) with the “TwoSampleMR” packages. The R code was provided in S1 File.

## Results

After primary screening, 30 SNPs for iron, 60 for ferritin, 29 for transferrin saturation and 33 for TIBC were selected. Among them, rs1260326, rs12979144 and rs12807014 for ferritin were removed because of the highly association with alcohol consumption. For transferrin saturation, rs4712972 was removed due to the highly association with body mass index. No outlier was found by MR PRESSO test for the four iron status biomarkers (S2 Table). Finally, we adopted 30 SNPs for iron, 57 for ferritin, 28 for transferrin saturation and 33 for TIBC as IVs. The information of SNPs as IVs and the corresponding information in outcome dataset are presented in S1 and S3 Tables.

**Fig 2:**
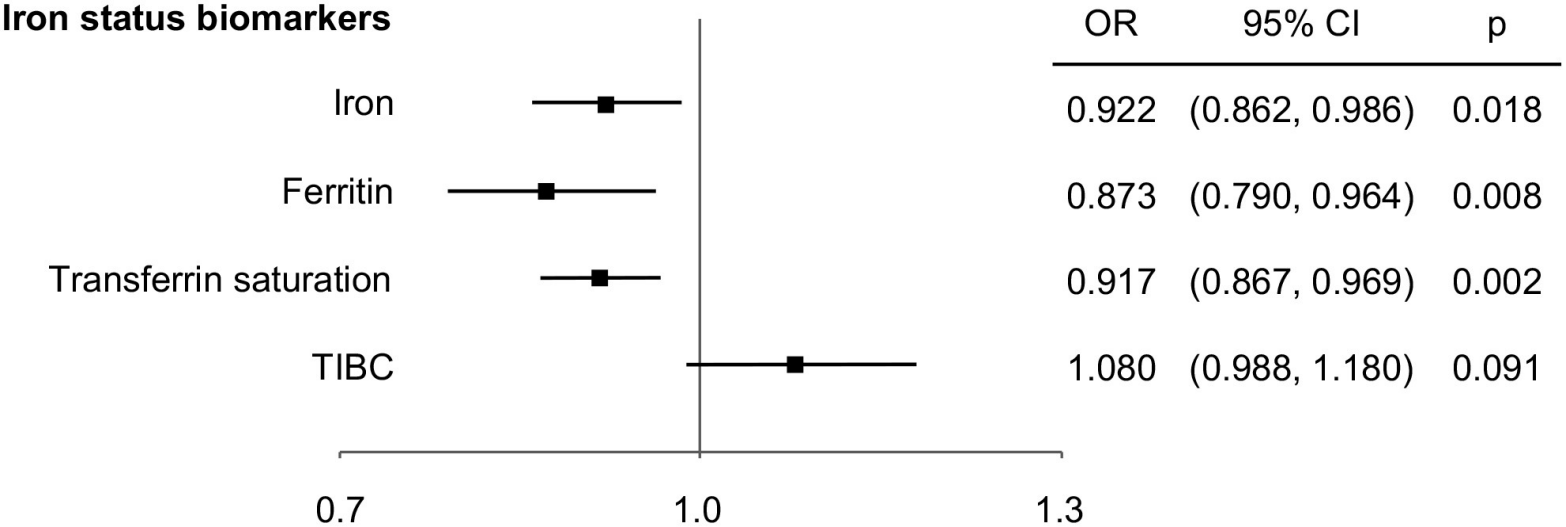
Association of genetically predicted four iron status biomarkers (per 1 SD increment) and the risk of anxiety disorders. OR, odd ratios; CI, confidence interval; TIBC, total iron-binding capacity.

**Fig 3:**
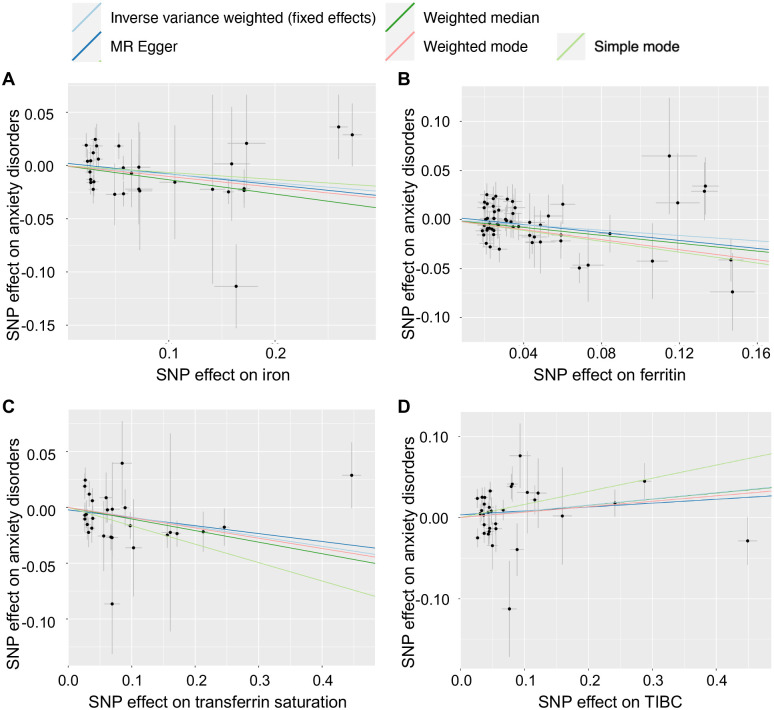
Scatter plots for the causal effects of serum iron (A), ferritin (B), transferrin (C) and TIBC (D) associated SNPs on anxiety disorders, using five different mendelian randomization analysis methods. TIBC, total iron-binding capacity.

The results of MR analysis are presented in Table 1. IVW analysis revealed that increased serum iron, ferritin and transferrin saturation significantly associated with decreased risk of anxiety disorders. The OR of anxiety disorders per 1 SD unit increment of serum iron, log-transformed ferritin and transferrin saturation were 0.922 (95% confidence interval (CI) 0.862–0.986; p = 0.018; power = 59.8%), 0.873 (95% CI 0.790–0.964; p = 0.008; power = 74.2%) and 0.917 (95% CI 0.867–0.969; p = 0.002; power = 75.5%), respectively (Fig 2, Table 1). The additional methods showed similar effects with consistent direction. Among them, weighted median and weighted mode revealed significant effect (p<0.05) as the IVW analyses did. While the effect of 1 SD unit increased TIBC on anxiety disorders was not statically significant (OR 1.080; 95% CI 0.988–1.180; p = 0.091; power = 49.9%). Fig 3 shows the scatter plots of MR analysis.

**Table 1:** Results of Mendelian randomization analysis of iron status biomarkers on anxiety disorders.

Exposure	Methods	β	SE	p	Power	Cochran’s Q p-value	MR Egger Intercept	Intercept p-value
**Iron (nSNP = 30)**	Inverse variance weighted	-0.082	0.034	**0.018**	59.8%	0.058	0.002	0.630
	MR Egger	-0.089	0.061	0.156				
	Weighted median	-0.134	0.051	0.008				
	Simple mode	-0.064	0.109	0.564				
	Weighted mode	-0.105	0.046	0.032				
**Ferritin (nSNP = 57)**	Inverse variance weighted	-0.136	0.051	**0.008**	74.2%	0.078	0.003	0.460
	MR Egger	-0.203	0.107	0.063				
	Weighted median	-0.201	0.085	0.018				
	Simple mode	-0.277	0.177	0.123				
	Weighted mode	-0.257	0.126	0.047				
**Transferrin saturation (nSNP = 28)**	Inverse variance weighted	-0.087	0.028	**0.002**	75.5%	0.161	-0.002	0.633
	MR Egger	-0.071	0.047	0.142				
	Weighted median	-0.104	0.042	0.013				
	Simple mode	-0.165	0.065	0.018				
	Weighted mode	-0.092	0.031	0.006				
**TIBC (nSNP = 33)**	Inverse variance weighted	0.077	0.045	0.091	49.9%	0.002	0.003	0.522
	MR Egger	0.048	0.064	0.459				
	Weighted median	0.076	0.054	0.160				
	Simple mode	0.162	0.115	0.169				
	Weighted mode	0.067	0.041	0.115				

nSNP, the number of SNPs selected as instrumental variables; SE, standard error; TIBC, total iron-binding capacity.

In addition, pleiotropy was not found for all the results (p>0.05 and the intercept trending to 0 in MR Egger intercept test). All the analyses showed low heterogeneity (p>0.05), except the one for TIBC effect (Table 1). Therefore, random effects model of IVW method was applied for the analysis of TIBC and fixed effects model for the other three iron status biomarkers. The forest plots, leave-one-out plots and funnel plots are available in S1–S3 Figs, which indicate that no individual SNP significantly affect the results.

## Discussion

In the present study, we assessed the causal correlation between four iron status biomarkers and the risk of anxiety disorders using MR analysis. We found that serum iron, ferritin and transferrin saturation were negatively correlated with the risk of anxiety disorders at genetically predicted level. It indicated that decreased systemic iron level contributed to the incidence of anxiety disorders.

Previous observational studies have researched the association of iron or related biomarkers with anxiety disorders. A comparative study found that serum level of iron was significantly higher in the generalized anxiety disorder patients than control [6]. Meanwhile, about eighty percent patients suffering mostly from anxiety disorders showed iron deficiency [17]. As the common consequence of iron deficiency, iron deficiency anemia (IDA) was found to be related to greater incidence and risk of anxiety disorders, which could be mitigated by iron supplementation [18]. Findings in these studies are in line with our results in present study that decreased circulating iron level gave rise to the elevated risk of anxiety disorders. As to TIBC, its correlation with anxiety disorders showed no statistical significance in our results. While the effect revealed inverse direction (β>0) compared to other three iron status biomarkers (β<0). Since TIBC is negatively responding to the circulating iron level and determined by total transferrin, the inverse effect is comprehensible. Inverse correlation of serum ferritin with anxiety symptom was also observed in female adolescent according to a pilot study [19].

To our knowledge, there was only one study using MR method to assess the link between iron status and mental disorders. They mentioned that no effective estimate was found in the relation between iron status biomarkers and anxiety disorders [20]. The GWAS data they used in the research were from a meta-analysis in 2014 with 48972 individuals [21]. And only 3 SNPs were adopted as IVs in the final analysis. In our study, we extracted IVs from the summary-level data of a recent meta-analysis of three GWAS for iron status biomarkers [7], involving larger sample size (131471 to 246139). In addition, they found forty-six new loci associated with iron status biomarkers, many of which were selected as IVs in our study under strict filter criteria (S1 Table). So, in our analysis more SNPs were selected in the MR analysis other than three ones. The inconsistence between the previous study mentioned above and the present study may be partially attributed to the underpowered sample size and IVs. Thus, our findings may be more comprehensive for the exploration of iron status effect on anxiety disorders.

The pathological mechanisms of iron status affecting anxiety disorders may involve the brain development and neurotransmitters. During early stage of brain development, researchers found that hippocampus was quite reactive in response to iron deficiency [22]. Iron deficiency affects the methylation of DNA and gene expression in hippocampus [23, 24]. On the other hand, iron is an important cofactor of many enzymes, including the ones that involved in neurotransmitters synthesis and metabolism. Lack of iron influences the activity of tyrosine hydroxylase for dopamine synthesis and tryptophan hydroxylase for serotonin synthesis [25]. Some studies revealed that monoamine oxidase showed lower activity in condition of iron deficiency anemia [26, 27]. The activities of GABA shunt enzymes such as glutamate dehydrogenase and GABA transaminase were also declined in iron deficient rats [28]. Moreover, iron deficiency may lead to elevation of extracellular dopamine and norepinephrine, along with reduction of dopamine receptor 1/2 receptors and all monoamine transporters [29, 30]. Although most of the studies mentioned above were performed on rodents, they still provided valuable rationale to the involvement of iron in the incidence of anxiety disorders.

There were some limitations in the present study. Firstly, the association of TIBC with anxiety disorders showed lack of significance, which may be caused by the underpower of the analysis (power = 49.9%). This limitation probably attributes to the inadequate sample size of cases for anxiety disorders in the dataset we used. Secondly, we used GWAS summary-level data as data sources, in which information about general factors like age and genders were not fully provided, making it unavailable to conduct stratified analyses and explore whether the effect of iron status biomarkers on anxiety disorders is non-linear or dose-response. What’s more, we cannot absolutely exclude a pleiotropic bias because the biological functions of some SNPs we adopted as IVs remain less understood. In consideration of that, we performed several sensitivity analyses to verify the reliability and robustness of the results. Lastly, our study was based on the European population, which limited the further application to other populations due to the different SNPs features like allele frequencies.

In conclusion, our present study explored the association of iron status biomarkers with anxiety disorders at genetic level by MR study using the large GWAS summary statistics. We found that decreased iron status including iron storage and transport elevated the risk of anxiety disorders. Maintaining appropriate serum iron level may contribute to the prevention and clinical intervention of anxiety disorders.

## Supporting information

S1 Table Characteristics of SNPs selected as IVs. (DOCX)

S2 Table Original results of MR PRESSO test. (DOCX)

S3 Table Characteristics of IVs in outcome dataset. (DOCX)

S1 Fig Forest plots for the effects of serum iron (A), ferritin (B), transferrin (C) and TIBC (D) associated SNPs on anxiety disorders.
(TIF)

S2 Fig Leave-one-out plots for the effects of serum iron (A), ferritin (B), transferrin (C) and TIBC (D) associated SNPs on anxiety disorders.
(TIF)

S3 Fig Funnel plots for the effects of serum iron (A), ferritin (B), transferrin (C) and TIBC (D) associated SNPs on anxiety disorders.
(TIF)

S1 File R code. (DOCX)

S2 File PRISMA 2020 checklist. (DOCX)

## References

[1] GBD 2019 Diseases and Injuries Collaborators. Global burden of 369 diseases and injuries in 204 countries and territories, 1990–2019: a systematic analysis for the Global Burden of Disease Study 2019. Lancet. 2020;396: 1204–1222. doi: 10.1016/S0140-6736(20)30925-9 33069326 PMC7567026

[2] Thibaut F. Anxiety disorders: a review of current literature. Dialogues Clin Neurosci. 2017;19: 87–88. doi: 10.31887/DCNS.2017.19.2/fthibaut 28867933 PMC5573565

[3] Deng MG, Liu F, Liang Y, Chen Y, Nie JQ, Chai C, et al. Associations of serum zinc, copper, and selenium with sleep disorders in the American adults: Data from NHANES 2011–2016. J Affect Disord. 2023;323: 378–385. https://10.1016/j.jad.2022.11.088 36464094 10.1016/j.jad.2022.11.088

[4] Deng MG, Cui HT, Nie JQ, Liang Y, Chai C. Genetic association between circulating selenium level and the risk of schizophrenia in the European population: A two-sample Mendelian randomization study. Front Nutr. 2022;9: 969887. https://10.3389/fnut.2022.969887 36082036 10.3389/fnut.2022.969887 PMC9445556

[5] Młyniec K, Gaweł M, Doboszewska U, Starowicz G, Nowak G. The role of elements in anxiety. Vitam Horm. 2017;103: 295–326. doi: 10.1016/bs.vh.2016.09.002 28061974

[6] Islam MR, Ahmed MU, Mitu SA, Islam MS, Rahman GK, Qusar MM, et al. Comparative analysis of serum zinc, copper, manganese, iron, calcium, and magnesium level and complexity of interelement relations in generalized anxiety disorder patients. Biol Trace Elem Res. 2013;154: 21–27. doi: 10.1007/s12011-013-9723-7 23754591

[7] Bell S, Rigas AS, Magnusson MK, Ferkingstad E, Allara E, Bjornsdottir G, et al. A genome-wide meta-analysis yields 46 new loci associating with biomarkers of iron homeostasis. Commun Biol. 2021;4: 156. doi: 10.1038/s42003-020-01575-z 33536631 PMC7859200

[8] Winter WE, Bazydlo LA, Harris NS. The molecular biology of human iron metabolism. Lab Med. 2017;45: 92–102. doi: 10.1309/lmf28s2gimxnwhmm 24868988

[9] Okan S, Caglıyan Turk A, Sıvgın H, Ozsoy F, Okan F. Association of ferritin levels with depression, anxiety, sleep quality, and physical functioning in patients with fibromyalgia syndrome: a cross-sectional study. Croat Med J. 2019;60: 515–520. doi: 10.3325/cmj.2019.60.515 31894917 PMC6952899

[10] Tsai Z, Shah N, Tahir U, Mortaji N, Owais S, Perreault M, et al. Dietary interventions for perinatal depression and anxiety: a systematic review and meta-analysis of randomized controlled trials. Am J Clin Nutr. 2023;117: 1130–1142. doi: 10.1016/j.ajcnut.2023.03.025 37019362

[11] Burgess S, Small DS, Thompson SG. A review of instrumental variable estimators for Mendelian randomization. Stat Methods Med Res. 2017;26: 2333–2355. doi: 10.1177/0962280215597579 26282889 PMC5642006

[12] Lawlor DA, Harbord RM, Sterne JA, Timpson N, Davey Smith G. Mendelian randomization: using genes as instruments for making causal inferences in epidemiology. Stat Med 2008;27: 1133–1163. doi: 10.1002/sim.3034 17886233

[13] Moylan S, Jacka FN, Pasco JA, Berk M. How cigarette smoking may increase the risk of anxiety symptoms and anxiety disorders: a critical review of biological pathways. Brain Behav. 2013;3: 302–326. doi: 10.1002/brb3.137 23785661 PMC3683289

[14] Skogen JC, Harvey SB, Henderson M, Stordal E, Mykletun A. Anxiety and depression among abstainers and low-level alcohol consumers. The Nord-Trøndelag Health Study. Addiction. 2009;104: 1519–1529. doi: 10.1111/j.1360-0443.2009.02659.x 19686521

[15] de Wit L, Have MT, Cuijpers P, de Graaf R. Body Mass Index and risk for onset of mood and anxiety disorders in the general population: Results from the Netherlands Mental Health Survey and Incidence Study-2 (NEMESIS-2). BMC Psychiatry. 2022;22: 522. doi: 10.1186/s12888-022-04077-w 35918662 PMC9344769

[16] Papadimitriou N, Dimou N, Tsilidis KK, Banbury B, Martin RM, Lewis SJ, et al. Physical activity and risks of breast and colorectal cancer: a Mendelian randomisation analysis. Nat Commun. 2020;11: 597. doi: 10.1038/s41467-020-14389-8 32001714 PMC6992637

[17] Kassir A. Iron deficiency: A diagnostic and therapeutic perspective in psychiatry. Encephale. 2017;43: 85–89. doi: 10.1016/j.encep.2016.08.002 27644916

[18] Lee HS, Chao HH, Huang WT, Chen SC, Yang HY. Psychiatric disorders risk in patients with iron deficiency anemia and association with iron supplementation medications: a nationwide database analysis. BMC Psychiatry. 2020;20: 216. doi: 10.1186/s12888-020-02621-0 32393355 PMC7216322

[19] Abbas M, Gandy K, Salas R, Devaraj S, Calarge CA. Iron deficiency and internalizing symptom severity in unmedicated adolescents: a pilot study. Psychol Med. 2023;53: 2274–2284. doi: 10.1017/S0033291721004098 34911595

[20] Qiu J, Lian F, Fang X. Iron status and mental disorders: a Mendelian randomization study. Front Nutr. 2022;9:1084860. doi: 10.3389/fnut.2022.1084860 36590208 PMC9797506

[21] Benyamin B, Esko T, Ried JS, Radhakrishnan A, Vermeulen SH, Traglia M, et al. Novel loci affecting iron homeostasis and their effects in individuals at risk for hemochromatosis. Nat Commun. 2014;5: 4926. doi: 10.1038/ncomms5926 25352340 PMC4215164

[22] Lozoff B, Georgieff MK. Iron deficiency and brain development. Semin Pediatr Neurol. 2006;13: 158–165. doi: 10.1016/j.spen.2006.08.004 17101454

[23] Lien YC, Condon DE, Georgieff MK, Simmons RA, Tran PV. 2019. Dysregulation of neuronal genes by fetal-neonatal iron deficiency anemia is associated with altered DNA methylation in the rat hippocampus. Nutrients. 2019;11: 1191. doi: 10.3390/nu11051191 31137889 PMC6566599

[24] Burns M, Amaya A, Bodi C, Ge Z, Bakthavatchalu V, Ennis K, et al. Helicobacter pylori infection and low dietary iron alter behavior, induce iron deficiency anemia, and modulate hippocampal gene expression in female C57BL/6 mice. PLoS One. 2017;12: e0173108. doi: 10.1371/journal.pone.0173108 28355210 PMC5371292

[25] Youdim MB, Green AR. Iron deficiency and neurotransmitter synthesis and function. Proc Nutr Soc. 1987;37: 173–179. doi: 10.1079/pns19780022 30090

[26] Symes AL, Sourkes TL, Youdim MB, Gregoriadis G, Birnbaum H. Decreased monoamine oxidase activity in liver of iron-deficient rats. Can J Biochem. 1969;47: 999–1002. doi: 10.1139/o69-160 5353933

[27] Youdim MB, Grahame-Smith DG, Woods HF. Some properties of human platelet monoamine oxidase in iron-deficiency anaemia. Clin Sci Mol Med. 1976;50: 479–485. doi: 10.1042/cs0500479 6185

[28] Taneja V, Mishra KP, Agarwal KN. Effect of maternal iron deficiency on GABA shunt pathway of developing rat brain. India J Exp Biol. 1990;28: 466–469. 2401520

[29] Beard JL, Connor JR. Iron status and neural functioning. Annu Rev Nutr. 2003;23: 41–58. doi: 10.1146/annurev.nutr.23.020102.075739 12704220

[30] Felt BT, Beard JL, Schallert T, Shao J, Aldridge JW, Connor JR, et al. Persistent neurochemical and behavioral abnormalities in adulthood despite early iron supplementation for perinatal iron deficiency anemia in rats. Behav Brain Res. 2006;171: 261–270. doi: 10.1016/j.bbr.2006.04.001 16713640 PMC1851886

