# Supplementary material for: The causal effect of iron status on risk of anxiety disorders: A two-sample Mendelian randomization study
Source: PLoS One. 2024 Mar 28;19(3):e0300143. doi: 10.1371/journal.pone.0300143 (PMC10977787; doi:10.1371/journal.pone.0300143)
Supplement: S1 Table — (DOCX) [file pone.0300143.s001.docx]

**S1 Table. Characteristics of SNPs selected as instrumental variables (IVs).**

| Exposure | SNP | Chr | Pos | EAF | Gene | EA | OA | R^2^ | F value | Exposure | | | Outcome | | |
| --- | --- | --- | --- | --- | --- | --- | --- | --- | --- | --- | --- | --- | --- | --- | --- |
|  |  |  |  |  |  |  |  |  |  | Beta | SE | P | Beta | SE | P |
| Iron | rs10421599 | 19 | 35767933 | 0.23 | USF2 | A | G | 2.24E-04 | 36.587 | -0.030 | 0.005 | 1.46E-09 | 0.015 | 0.014 | 0.260 |
| Iron | rs10822143 ^b^ | 10 | 64934548 | 0.49 | RP11-144G16.1 | C | T | 2.10E-04 | 34.343 | -0.024 | 0.004 | 4.62E-09 | 0.004 | 0.011 | 0.723 |
| Iron | rs114708114 | 6 | 28426244 | 0.03 | OR2E1P | T | C | 8.43E-04 | 137.936 | 0.141 | 0.012 | 7.52E-32 | -0.022 | 0.089 | 0.801 |
| Iron | rs116009877 | 6 | 25715657 | 0.08 | ZFP57 | A | G | 5.56E-03 | 913.752 | 0.260 | 0.009 | 1.00E-200 | 0.036 | 0.030 | 0.233 |
| Iron | rs116169498 | 6 | 26104664 | 0.02 | HIST1H1T | G | C | 5.38E-04 | 88.093 | 0.159 | 0.017 | 6.24E-21 | 0.002 | 0.054 | 0.978 |
| Iron | rs117718169 | 22 | 37359621 | 0.05 | LL22NC01-81G9.3 | T | C | 2.69E-04 | 44.002 | -0.072 | 0.011 | 3.28E-11 | 0.002 | 0.042 | 0.969 |
| Iron | rs117753190 | 17 | 67136325 | 0.04 | ABCA6 | G | C | 2.41E-04 | 39.447 | 0.073 | 0.012 | 3.37E-10 | -0.024 | 0.056 | 0.667 |
| Iron | rs12206204 | 6 | 26116982 | 0.02 | HIST1H2BC | T | C | 5.75E-04 | 94.139 | 0.173 | 0.018 | 2.94E-22 | 0.021 | 0.046 | 0.648 |
| Iron | rs12633819 | 3 | 66448307 | 0.34 | LRIG1 | G | A | 2.36E-04 | 38.548 | 0.026 | 0.004 | 5.34E-10 | -0.006 | 0.013 | 0.624 |
| Iron | rs12718598 ^a^ | 7 | 50428445 | 0.49 | IKZF1 | C | T | 2.68E-04 | 43.771 | 0.027 | 0.004 | 3.69E-11 | -0.016 | 0.011 | 0.162 |
| Iron | rs12730935 ^b^ | 1 | 154420087 | 0.40 | IL6R | A | G | 2.58E-04 | 42.235 | 0.026 | 0.004 | 8.09E-11 | -0.013 | 0.012 | 0.283 |
| Iron | rs12975762 | 19 | 35950523 | 0.29 | FFAR2 | G | A | 2.77E-04 | 45.288 | -0.029 | 0.004 | 1.70E-11 | 0.022 | 0.013 | 0.092 |
| Iron | rs13007705 ^a^ | 2 | 239069196 | 0.45 | ERFE | T | C | 3.02E-04 | 49.474 | 0.029 | 0.004 | 2.01E-12 | 0.012 | 0.012 | 0.302 |
| Iron | rs140393761 | 6 | 25737913 | 0.02 | HIST1H2APS1 | G | A | 3.24E-04 | 52.997 | 0.106 | 0.015 | 3.34E-13 | -0.016 | 0.053 | 0.770 |
| Iron | rs145496147 | 6 | 26099201 | 0.01 | HIST1H4C | T | C | 3.84E-04 | 62.832 | 0.163 | 0.021 | 2.25E-15 | -0.114 | 0.039 | 0.004 |
| Iron | rs1799945 ^a^ | 6 | 26091179 | 0.15 | HFE | G | C | 5.19E-03 | 853.491 | 0.171 | 0.006 | 1.26E-187 | -0.022 | 0.018 | 0.225 |
| Iron | rs1800562 ^a^ | 6 | 26093141 | 0.08 | HFE | A | G | 5.56E-03 | 913.752 | 0.273 | 0.009 | 1.00E-200 | 0.029 | 0.030 | 0.328 |
| Iron | rs1958078 | 14 | 70354858 | 0.16 | SMOC1 | A | C | 1.90E-04 | 31.077 | -0.032 | 0.006 | 2.48E-08 | 0.018 | 0.021 | 0.376 |
| Iron | rs35945185 ^a,b^ | 1 | 66147846 | 0.37 | LEPR | A | G | 3.33E-04 | 54.518 | 0.031 | 0.004 | 1.54E-13 | 0.025 | 0.011 | 0.029 |
| Iron | rs4774514 | 15 | 45374055 | 0.05 | SORD | T | C | 2.16E-04 | 35.27 | -0.049 | 0.008 | 2.87E-09 | 0.027 | 0.029 | 0.351 |
| Iron | rs4854760 | 3 | 133498741 | 0.34 | SRPRB | G | A | 8.74E-04 | 143.07 | 0.053 | 0.004 | 5.67E-33 | 0.018 | 0.012 | 0.136 |
| Iron | rs563027675 | 22 | 37822836 | 0.02 | ELFN2 | G | C | 1.87E-04 | 30.625 | -0.072 | 0.013 | 3.13E-08 | 0.022 | 0.033 | 0.502 |
| Iron | rs56912861 | 22 | 37511637 | 0.34 | SCUBE1 | A | G | 3.94E-04 | 64.391 | -0.034 | 0.004 | 1.02E-15 | -0.006 | 0.011 | 0.592 |
| Iron | rs7385804 | 7 | 100235970 | 0.37 | TFR2 | C | A | 1.15E-03 | 187.837 | -0.057 | 0.004 | 9.42E-43 | -0.002 | 0.011 | 0.843 |
| Iron | rs7837764 | 8 | 23375702 | 0.38 | SLC25A37 | C | G | 1.82E-04 | 29.724 | 0.022 | 0.004 | 4.98E-08 | 0.019 | 0.012 | 0.101 |
| Iron | rs855791 a | 22 | 37462936 | 0.44 | TMPRSS6 | A | G | 5.56E-03 | 913.751 | -0.171 | 0.006 | 1.00E-200 | -0.024 | 0.012 | 0.045 |
| Iron | rs9265878 | 6 | 31313033 | 0.27 | HLA-B | A | G | 2.02E-04 | 32.976 | -0.027 | 0.005 | 9.33E-09 | -0.004 | 0.013 | 0.732 |
| Iron | rs9295767 | 6 | 28182450 | 0.04 | TOB2P1 | A | G | 2.92E-04 | 47.726 | -0.065 | 0.009 | 4.90E-12 | 0.007 | 0.032 | 0.822 |
| Iron | rs9399136 ^a,b^ | 6 | 135421176 | 0.25 | HBS1L | C | T | 9.78E-04 | 160.091 | 0.057 | 0.005 | 1.08E-36 | -0.027 | 0.012 | 0.025 |
| Iron | rs9610638 b | 22 | 37470604 | 0.43 | KCTD17 | T | C | 5.56E-03 | 913.751 | -0.156 | 0.005 | 1.00E-200 | -0.025 | 0.011 | 0.029 |
| Ferritin | rs10263500 | 7 | 30784551 | 0.48 | INMT | T | C | 1.73E-04 | 42.637 | 0.023 | 0.003 | 6.59E-11 | -0.004 | 0.011 | 0.741 |
| Ferritin | rs10750215 | 11 | 122505344 | 0.39 | RP11-266E8.2 | T | G | 1.23E-04 | 30.321 | 0.021 | 0.004 | 3.66E-08 | 0.016 | 0.012 | 0.165 |
| Ferritin | rs10801913 ^a^ | 1 | 116214279 | 0.30 | VANGL1 | A | G | 1.62E-04 | 39.931 | 0.024 | 0.004 | 2.63E-10 | 0.010 | 0.012 | 0.431 |
| Ferritin | rs10804630 | 3 | 133997989 | 0.11 | KRT18P35 | C | T | 1.41E-04 | 34.77 | -0.034 | 0.006 | 3.71E-09 | 0.002 | 0.016 | 0.880 |
| Ferritin | rs11634990 | 15 | 78771186 | 0.16 | IREB2 | C | T | 1.34E-04 | 32.961 | -0.027 | 0.005 | 9.40E-09 | 0.005 | 0.015 | 0.758 |
| Ferritin | rs12419620 ^a^ | 11 | 2232553 | 0.17 | MIR4686 | G | T | 1.78E-04 | 43.914 | -0.031 | 0.005 | 3.43E-11 | 0.001 | 0.016 | 0.946 |
| Ferritin | rs1250244 | 2 | 216297796 | 0.27 | FN1 | G | C | 1.78E-04 | 43.842 | -0.025 | 0.004 | 3.56E-11 | -0.011 | 0.014 | 0.424 |
| Ferritin | rs12568930 | 1 | 22702231 | 0.18 | RP11-415K20.2 | C | T | 2.38E-04 | 58.653 | 0.035 | 0.005 | 1.88E-14 | 0.006 | 0.020 | 0.757 |
| Ferritin | rs12916360 | 15 | 65856805 | 0.13 | HACD3 | T | G | 1.28E-04 | 31.514 | -0.027 | 0.005 | 1.98E-08 | 0.006 | 0.013 | 0.667 |
| Ferritin | rs13253974 ^a^ | 8 | 23377910 | 0.32 | SLC25A37 | A | G | 1.81E-04 | 44.526 | 0.024 | 0.004 | 2.51E-11 | 0.021 | 0.012 | 0.086 |
| Ferritin | rs143922167 | 19 | 49403841 | 0.02 | NUCB1 | A | G | 2.64E-04 | 64.948 | 0.115 | 0.014 | 7.69E-16 | 0.065 | 0.059 | 0.275 |
| Ferritin | rs144861591 | 6 | 26072992 | 0.08 | ZFP57 | T | C | 1.55E-03 | 381.047 | 0.133 | 0.007 | 7.35E-85 | 0.034 | 0.029 | 0.242 |
| Ferritin | rs161044 | 12 | 51382232 | 0.06 | SLC11A2 | T | C | 3.34E-04 | 82.167 | 0.073 | 0.008 | 1.25E-19 | 0.047 | 0.037 | 0.211 |
| Ferritin | rs1694067 ^b^ | 5 | 53284241 | 0.37 | ARL15 | T | C | 1.44E-04 | 35.515 | -0.021 | 0.004 | 2.53E-09 | 0.025 | 0.012 | 0.029 |
| Ferritin | rs17050272 | 2 | 121306440 | 0.41 | AC073257.2 | A | G | 1.40E-04 | 34.561 | 0.021 | 0.004 | 4.13E-09 | -0.024 | 0.011 | 0.031 |
| Ferritin | rs17112021 | 10 | 101343726 | 0.24 | snoU13 | G | T | 1.54E-04 | 37.845 | 0.024 | 0.004 | 7.66E-10 | 0.014 | 0.014 | 0.334 |
| Ferritin | rs17476364 ^a^ | 10 | 71094504 | 0.11 | HK1 | C | T | 2.33E-04 | 57.392 | 0.043 | 0.006 | 3.57E-14 | -0.016 | 0.024 | 0.500 |
| Ferritin | rs17676097 | 17 | 9791549 | 0.33 | GLP2R | G | A | 2.29E-04 | 56.351 | -0.027 | 0.004 | 6.06E-14 | -0.010 | 0.012 | 0.426 |
| Ferritin | rs1799945 ^a^ | 6 | 26091179 | 0.15 | HFE | G | C | 5.54E-04 | 136.552 | 0.059 | 0.005 | 1.51E-31 | -0.022 | 0.018 | 0.225 |
| Ferritin | rs1800562 ^a^ | 6 | 26093141 | 0.08 | HFE | A | G | 1.54E-03 | 379.206 | 0.133 | 0.007 | 1.85E-84 | 0.029 | 0.030 | 0.328 |
| Ferritin | rs1894692 | 1 | 169467654 | 0.02 | RP1-206D15.6 | G | A | 6.65E-04 | 163.85 | 0.147 | 0.011 | 1.63E-37 | 0.074 | 0.040 | 0.063 |
| Ferritin | rs199138 | 15 | 45387550 | 0.07 | DUOX2 | A | G | 2.11E-03 | 519.655 | -0.146 | 0.006 | 5.02E-115 | -0.041 | 0.022 | 0.060 |
| Ferritin | rs2008598 | 6 | 131145306 | 0.31 | SMLR1 | G | A | 1.25E-04 | 30.74 | -0.021 | 0.004 | 2.95E-08 | 0.000 | 0.015 | 0.995 |
| Ferritin | rs2529440 ^a^ | 7 | 30511794 | 0.46 | NOD1 | T | C | 3.97E-04 | 97.811 | -0.035 | 0.003 | 4.60E-23 | 0.008 | 0.011 | 0.502 |
| Ferritin | rs2595582 | 20 | 3630107 | 0.40 | ATRN | A | G | 1.25E-04 | 30.876 | -0.020 | 0.004 | 2.75E-08 | 0.016 | 0.012 | 0.188 |
| Ferritin | rs28715334 | 14 | 23635213 | 0.19 | SLC7A8 | T | G | 1.35E-04 | 33.222 | 0.025 | 0.004 | 8.22E-09 | 0.008 | 0.016 | 0.614 |
| Ferritin | rs34523089 | 17 | 56436109 | 0.16 | RNF43 | T | C | 8.64E-04 | 212.923 | 0.069 | 0.005 | 3.16E-48 | -0.049 | 0.015 | 0.001 |
| Ferritin | rs35107257 | 5 | 68805616 | 0.04 | OCLN | A | G | 1.24E-04 | 30.54 | 0.043 | 0.008 | 3.27E-08 | -0.003 | 0.030 | 0.921 |
| Ferritin | rs36184164 | 6 | 43781092 | 0.12 | RP11-344J7.3 | G | T | 1.92E-04 | 47.184 | 0.036 | 0.005 | 6.46E-12 | 0.012 | 0.020 | 0.545 |
| Ferritin | rs370631 | 6 | 29732744 | 0.29 | XXbac-BPG170G13.31 | C | T | 1.79E-04 | 44.008 | -0.026 | 0.004 | 3.27E-11 | -0.024 | 0.015 | 0.101 |
| Ferritin | rs45520632 | 19 | 49426488 | 0.05 | NUCB1 | C | T | 6.99E-04 | 172.246 | 0.106 | 0.008 | 2.39E-39 | -0.042 | 0.039 | 0.271 |
| Ferritin | rs4789111 | 17 | 72940481 | 0.16 | OTOP3 | T | C | 2.04E-04 | 50.284 | 0.034 | 0.005 | 1.33E-12 | -0.018 | 0.018 | 0.294 |
| Ferritin | rs4808802 ^a^ | 19 | 18577873 | 0.22 | ELL | C | G | 1.78E-04 | 43.92 | 0.028 | 0.004 | 3.42E-11 | -0.030 | 0.014 | 0.026 |
| Ferritin | rs4841429 ^a^ | 8 | 10568529 | 0.09 | RP1L1 | G | A | 3.56E-04 | 87.551 | 0.060 | 0.006 | 8.21E-21 | 0.016 | 0.020 | 0.439 |
| Ferritin | rs4938939 ^a^ | 11 | 60160838 | 0.30 | MS4A14 | A | G | 1.43E-04 | 35.177 | 0.022 | 0.004 | 3.01E-09 | -0.009 | 0.013 | 0.512 |
| Ferritin | rs532436 | 9 | 136149830 | 0.18 | ABO | A | G | 6.18E-04 | 152.273 | -0.059 | 0.005 | 5.52E-35 | 0.016 | 0.014 | 0.255 |
| Ferritin | rs55778511 | 15 | 45177748 | 0.06 | SORD2P | G | T | 1.96E-04 | 48.261 | -0.053 | 0.008 | 3.73E-12 | -0.004 | 0.022 | 0.868 |
| Ferritin | rs56206139 | 20 | 39108705 | 0.08 | LINC01728 | C | A | 1.95E-04 | 48.09 | 0.046 | 0.007 | 4.07E-12 | -0.018 | 0.031 | 0.568 |
| Ferritin | rs590097 | 2 | 111934107 | 0.35 | BCL2L11 | T | G | 1.21E-04 | 29.744 | -0.020 | 0.004 | 4.93E-08 | -0.006 | 0.012 | 0.603 |
| Ferritin | rs6059696 | 20 | 30095904 | 0.16 | RP11-101E14.3 | G | C | 2.59E-04 | 63.781 | -0.037 | 0.005 | 1.39E-15 | 0.007 | 0.014 | 0.603 |
| Ferritin | rs62074125 | 17 | 44852612 | 0.25 | WNT3 | C | A | 1.21E-04 | 29.8 | 0.021 | 0.004 | 4.79E-08 | -0.011 | 0.013 | 0.408 |
| Ferritin | rs6760824 | 2 | 29178319 | 0.29 | TOGARAM2 | A | C | 2.63E-04 | 64.743 | 0.032 | 0.004 | 8.53E-16 | -0.021 | 0.013 | 0.111 |
| Ferritin | rs6822746 | 4 | 56257107 | 0.35 | SRD5A3-AS1 | A | G | 1.38E-04 | 34 | -0.021 | 0.004 | 5.51E-09 | -0.001 | 0.012 | 0.914 |
| Ferritin | rs7068127 | 10 | 52606488 | 0.42 | A1CF | G | A | 1.29E-04 | 31.821 | 0.020 | 0.003 | 1.69E-08 | 0.012 | 0.012 | 0.297 |
| Ferritin | rs708686 ^a^ | 19 | 5840619 | 0.27 | FUT6 | T | C | 2.38E-04 | 58.571 | -0.031 | 0.004 | 1.96E-14 | 0.000 | 0.012 | 0.986 |
| Ferritin | rs71537957 | 7 | 150063843 | 0.27 | REPIN1 | T | C | 1.39E-04 | 34.17 | 0.023 | 0.004 | 5.05E-09 | -0.028 | 0.012 | 0.019 |
| Ferritin | rs72606621 | 8 | 87175491 | 0.27 | ATP6V0D2 | A | G | 1.23E-04 | 30.285 | 0.021 | 0.004 | 3.73E-08 | -0.009 | 0.012 | 0.440 |
| Ferritin | rs735831 | 2 | 190398497 | 0.06 | SLC40A1 | G | T | 1.62E-04 | 39.924 | 0.049 | 0.008 | 2.64E-10 | -0.023 | 0.032 | 0.476 |
| Ferritin | rs7596205 | 2 | 190428193 | 0.11 | SLC40A1 | A | G | 8.78E-04 | 216.361 | 0.084 | 0.006 | 5.62E-49 | -0.014 | 0.019 | 0.451 |
| Ferritin | rs75965181 | 1 | 22584002 | 0.02 | MIR4418 | A | T | 4.55E-04 | 111.93 | -0.119 | 0.011 | 3.70E-26 | -0.017 | 0.051 | 0.737 |
| Ferritin | rs7865362 | 9 | 33117965 | 0.37 | B4GALT1 | T | C | 1.88E-04 | 46.27 | 0.025 | 0.004 | 1.03E-11 | -0.015 | 0.012 | 0.199 |
| Ferritin | rs855791 ^a^ | 22 | 37462936 | 0.44 | TMPRSS6 | A | G | 6.55E-04 | 161.214 | -0.044 | 0.003 | 6.14E-37 | -0.024 | 0.012 | 0.045 |
| Ferritin | rs859788 | 8 | 109227570 | 0.45 | EIF3E | G | A | 1.21E-04 | 29.887 | -0.019 | 0.003 | 4.58E-08 | 0.012 | 0.011 | 0.304 |
| Ferritin | rs9512463 | 13 | 27528519 | 0.23 | RP11-545M8.4 | T | C | 1.43E-04 | 35.107 | 0.025 | 0.004 | 3.12E-09 | -0.001 | 0.013 | 0.928 |
| Ferritin | rs970079 | 5 | 98119864 | 0.51 | RGMB | G | A | 1.35E-04 | 33.201 | -0.020 | 0.003 | 8.31E-09 | -0.018 | 0.012 | 0.136 |
| Ferritin | rs9921222 ^a^ | 16 | 375782 | 0.54 | AXIN1 | C | T | 2.06E-04 | 50.675 | 0.025 | 0.003 | 1.09E-12 | -0.001 | 0.011 | 0.928 |
| Ferritin | rs996347 ^a^ | 14 | 34410892 | 0.36 | EGLN3 | C | T | 7.35E-04 | 180.959 | 0.049 | 0.004 | 2.99E-41 | -0.006 | 0.012 | 0.629 |
| Transferrin saturation | rs10421599 | 19 | 35767933 | 0.23 | USF2 | A | G | 2.26E-04 | 29.736 | -0.030 | 0.006 | 4.95E-08 | 0.015 | 0.014 | 0.260 |
| Transferrin saturation | rs114708114 | 6 | 28426244 | 0.03 | OR2E1P | T | C | 1.06E-03 | 139.174 | 0.161 | 0.014 | 4.03E-32 | -0.022 | 0.089 | 0.801 |
| Transferrin saturation | rs115314215 | 6 | 27425598 | 0.04 | ZNF184 | T | C | 2.47E-04 | 32.486 | -0.056 | 0.010 | 1.20E-08 | 0.026 | 0.032 | 0.416 |
| Transferrin saturation | rs116272812 | 6 | 25452783 | 0.13 | CARMIL1 | C | T | 6.90E-03 | 913.749 | 0.246 | 0.008 | 1.00E-200 | -0.018 | 0.017 | 0.291 |
| Transferrin saturation | rs117718169 | 22 | 37359621 | 0.05 | LL22NC01-81G9.3 | T | C | 2.50E-04 | 32.876 | -0.069 | 0.012 | 9.82E-09 | 0.002 | 0.042 | 0.969 |
| Transferrin saturation | rs12478088 | 2 | 190423005 | 0.38 | SLC40A1 | G | A | 2.41E-04 | 31.753 | 0.026 | 0.005 | 1.75E-08 | 0.007 | 0.011 | 0.539 |
| Transferrin saturation | rs12633819 | 3 | 66448307 | 0.34 | LRIG1 | G | A | 2.76E-04 | 36.336 | 0.029 | 0.005 | 1.66E-09 | -0.006 | 0.013 | 0.624 |
| Transferrin saturation | rs12975762 | 19 | 35950523 | 0.29 | FFAR2 | G | A | 3.26E-04 | 42.876 | -0.032 | 0.005 | 5.83E-11 | 0.022 | 0.013 | 0.092 |
| Transferrin saturation | rs13007705 ^a^ | 2 | 239069196 | 0.45 | ERFE | T | C | 3.85E-04 | 50.656 | 0.033 | 0.005 | 1.10E-12 | 0.012 | 0.012 | 0.302 |
| Transferrin saturation | rs1799945 ^a^ | 6 | 26091179 | 0.15 | HFE | G | C | 6.90E-03 | 913.749 | 0.213 | 0.007 | 1.00E-200 | -0.022 | 0.018 | 0.225 |
| Transferrin saturation | rs1800562 ^a^ | 6 | 26093141 | 0.08 | HFE | A | G | 6.90E-03 | 913.749 | 0.447 | 0.015 | 1.00E-200 | 0.029 | 0.030 | 0.328 |
| Transferrin saturation | rs28487964 | 15 | 44784375 | 0.03 | CTDSPL2 | A | T | 2.33E-04 | 30.675 | -0.069 | 0.012 | 3.05E-08 | 0.087 | 0.045 | 0.054 |
| Transferrin saturation | rs35945185 ^b^ | 1 | 66147846 | 0.37 | LEPR | A | G | 2.45E-04 | 32.272 | 0.027 | 0.005 | 1.34E-08 | 0.025 | 0.011 | 0.029 |
| Transferrin saturation | rs4774514 | 15 | 45374055 | 0.05 | SORD | T | C | 4.04E-04 | 53.099 | -0.069 | 0.009 | 3.17E-13 | 0.027 | 0.029 | 0.351 |
| Transferrin saturation | rs56912861 | 22 | 37511637 | 0.34 | SCUBE1 | A | G | 4.88E-04 | 64.137 | -0.038 | 0.005 | 1.16E-15 | -0.006 | 0.011 | 0.592 |
| Transferrin saturation | rs62396224 | 6 | 26292926 | 0.05 | ZFP57 | G | A | 2.53E-04 | 33.321 | -0.060 | 0.010 | 7.81E-09 | -0.009 | 0.023 | 0.700 |
| Transferrin saturation | rs6456691 | 6 | 25709223 | 0.13 | ZFP57 | C | T | 1.30E-03 | 171.022 | 0.090 | 0.007 | 4.42E-39 | 0.000 | 0.017 | 0.984 |
| Transferrin saturation | rs6592965 | 7 | 50427982 | 0.46 | IKZF1 | A | G | 2.48E-04 | 32.673 | 0.026 | 0.005 | 1.09E-08 | -0.010 | 0.012 | 0.389 |
| Transferrin saturation | rs72838865 | 6 | 25671889 | 0.03 | SCGN | A | C | 4.72E-04 | 62.145 | 0.103 | 0.013 | 3.19E-15 | -0.036 | 0.044 | 0.406 |
| Transferrin saturation | rs7385804 | 7 | 100235970 | 0.37 | TFR2 | C | A | 1.28E-03 | 168.897 | -0.062 | 0.005 | 2.87E-39 | -0.002 | 0.011 | 0.843 |
| Transferrin saturation | rs74338506 | 6 | 112054302 | 0.03 | FYN | A | G | 2.34E-04 | 30.793 | -0.085 | 0.015 | 2.87E-08 | -0.040 | 0.038 | 0.294 |
| Transferrin saturation | rs7648210 | 3 | 195835839 | 0.24 | - | A | G | 3.73E-04 | 49.022 | 0.037 | 0.005 | 2.53E-12 | 0.019 | 0.013 | 0.156 |
| Transferrin saturation | rs7650925 ^b^ | 3 | 133529594 | 0.53 | SRPRB | T | G | 5.40E-04 | 71.017 | 0.039 | 0.005 | 3.54E-17 | -0.010 | 0.011 | 0.383 |
| Transferrin saturation | rs7837764 | 8 | 23375702 | 0.38 | SLC25A37 | C | G | 2.43E-04 | 31.915 | 0.026 | 0.005 | 1.61E-08 | 0.019 | 0.012 | 0.101 |
| Transferrin saturation | rs8177271 | 3 | 133482230 | 0.34 | TF | A | G | 2.97E-03 | 390.988 | -0.098 | 0.005 | 5.03E-87 | 0.016 | 0.012 | 0.178 |
| Transferrin saturation | rs855791 ^a^ | 22 | 37462936 | 0.44 | TMPRSS6 | A | G | 6.90E-03 | 913.749 | -0.171 | 0.006 | 1.00E-200 | -0.024 | 0.012 | 0.045 |
| Transferrin saturation | rs9399136 ^a,b^ | 6 | 135421176 | 0.25 | HBS1L | C | T | 1.30E-03 | 170.653 | 0.067 | 0.005 | 5.32E-39 | -0.027 | 0.012 | 0.025 |
| Transferrin saturation | rs9610638 b | 22 | 37470604 | 0.43 | KCTD17 | T | C | 6.90E-03 | 913.749 | -0.156 | 0.005 | 1.00E-200 | -0.025 | 0.011 | 0.029 |
| TIBC | rs1106735 | 3 | 133602009 | 0.13 | RAB6B | G | A | 8.63E-04 | 116.964 | 0.079 | 0.007 | 2.92E-27 | 0.038 | 0.016 | 0.014 |
| TIBC | rs112466891 | 3 | 133703833 | 0.19 | SLCO2A1 | C | A | 4.02E-04 | 54.406 | 0.045 | 0.006 | 1.63E-13 | 0.012 | 0.013 | 0.330 |
| TIBC | rs112727702 ^a^ | 19 | 50092002 | 0.24 | NOSIP | T | G | 4.31E-04 | 58.454 | 0.043 | 0.006 | 2.08E-14 | -0.021 | 0.014 | 0.132 |
| TIBC | rs116272812 | 6 | 25452783 | 0.13 | CARMIL1 | C | T | 6.70E-03 | 913.749 | -0.241 | 0.008 | 1.00E-200 | -0.018 | 0.017 | 0.291 |
| TIBC | rs12206077 | 6 | 29440741 | 0.28 | UBDP1 | A | G | 1.24E-03 | 167.952 | -0.067 | 0.005 | 2.07E-38 | -0.009 | 0.013 | 0.463 |
| TIBC | rs12976652 | 19 | 46396610 | 0.12 | MYPOP | C | T | 2.62E-04 | 35.5 | -0.045 | 0.007 | 2.55E-09 | -0.013 | 0.023 | 0.584 |
| TIBC | rs13008704 | 2 | 190387487 | 0.50 | AC013439.4 | C | T | 4.58E-04 | 61.996 | -0.037 | 0.005 | 3.44E-15 | 0.017 | 0.011 | 0.141 |
| TIBC | rs13084306 | 3 | 133917298 | 0.02 | RYK | A | C | 3.05E-04 | 41.278 | 0.104 | 0.016 | 1.32E-10 | 0.031 | 0.051 | 0.541 |
| TIBC | rs143530446 | 3 | 133685958 | 0.02 | SLCO2A1 | T | C | 8.29E-04 | 112.372 | -0.159 | 0.015 | 2.96E-26 | -0.002 | 0.060 | 0.974 |
| TIBC | rs174547 | 11 | 61570783 | 0.34 | FADS1 | C | T | 6.83E-04 | 92.547 | 0.046 | 0.005 | 6.57E-22 | 0.033 | 0.011 | 0.004 |
| TIBC | rs17580 ^a^ | 14 | 94847262 | 0.05 | SERPINA1 | A | T | 3.06E-04 | 41.481 | 0.076 | 0.012 | 1.19E-10 | -0.113 | 0.059 | 0.057 |
| TIBC | rs17767742 | 16 | 79749005 | 0.31 | LINC01229 | G | C | 2.66E-04 | 36.022 | 0.031 | 0.005 | 1.95E-09 | 0.005 | 0.012 | 0.701 |
| TIBC | rs1799945 ^a^ | 6 | 26091179 | 0.15 | HFE | G | C | 2.17E-03 | 294.879 | -0.116 | 0.007 | 4.29E-66 | -0.022 | 0.018 | 0.225 |
| TIBC | rs1800562 ^a^ | 6 | 26093141 | 0.08 | HFE | A | G | 6.70E-03 | 913.749 | -0.449 | 0.015 | 1.00E-200 | 0.029 | 0.030 | 0.328 |
| TIBC | rs1927693 | 6 | 25414846 | 0.39 | CARMIL1 | A | G | 4.32E-04 | 58.512 | 0.037 | 0.005 | 2.02E-14 | -0.020 | 0.012 | 0.096 |
| TIBC | rs199138 | 15 | 45387550 | 0.07 | DUOX2 | A | G | 6.44E-04 | 87.243 | 0.081 | 0.009 | 9.59E-21 | -0.041 | 0.022 | 0.060 |
| TIBC | rs2236252 | 20 | 17597531 | 0.17 | RRBP1 | T | C | 2.56E-04 | 34.614 | 0.036 | 0.006 | 4.02E-09 | 0.003 | 0.016 | 0.848 |
| TIBC | rs35570672 | 8 | 18272635 | 0.22 | NAT2 | T | C | 4.05E-04 | 54.912 | -0.043 | 0.006 | 1.26E-13 | 0.006 | 0.013 | 0.630 |
| TIBC | rs35769520 | 14 | 75255115 | 0.54 | YLPM1 | G | A | 2.38E-04 | 32.272 | 0.026 | 0.005 | 1.34E-08 | 0.025 | 0.012 | 0.031 |
| TIBC | rs469882 ^a^ | 1 | 91530432 | 0.20 | RPL5P6 | C | A | 3.08E-04 | 41.763 | -0.037 | 0.006 | 1.03E-10 | 0.009 | 0.014 | 0.515 |
| TIBC | rs4846335 | 1 | 221032881 | 0.11 | HLX-AS1 | A | C | 2.60E-04 | 35.27 | 0.045 | 0.007 | 2.87E-09 | -0.017 | 0.016 | 0.280 |
| TIBC | rs56195124 | 3 | 134112872 | 0.05 | KRT18P35 | A | G | 4.91E-04 | 66.527 | 0.088 | 0.011 | 3.45E-16 | -0.040 | 0.033 | 0.223 |
| TIBC | rs59950280 ^a^ | 4 | 3452345 | 0.34 | HGFAC | A | G | 3.16E-04 | 42.863 | 0.033 | 0.005 | 5.87E-11 | 0.009 | 0.013 | 0.513 |
| TIBC | rs6025 ^a^ | 1 | 169519049 | 0.02 | F5 | T | C | 2.34E-04 | 31.742 | -0.093 | 0.017 | 1.76E-08 | 0.076 | 0.040 | 0.055 |
| TIBC | rs62183592 | 2 | 190429752 | 0.08 | SLC40A1 | T | C | 2.25E-04 | 30.516 | 0.050 | 0.009 | 3.31E-08 | -0.035 | 0.030 | 0.244 |
| TIBC | rs72840508 | 6 | 25624400 | 0.03 | CARMIL1 | T | A | 6.25E-04 | 84.741 | -0.121 | 0.013 | 3.40E-20 | -0.030 | 0.043 | 0.484 |
| TIBC | rs7297861 | 12 | 121026828 | 0.10 | RPL11P5 | C | T | 2.56E-04 | 34.628 | 0.048 | 0.008 | 3.99E-09 | 0.008 | 0.017 | 0.642 |
| TIBC | rs7432894 | 3 | 195841644 | 0.31 | - | C | T | 4.03E-04 | 54.543 | -0.038 | 0.005 | 1.52E-13 | 0.025 | 0.013 | 0.051 |
| TIBC | rs8177257 | 3 | 133480337 | 0.03 | TF | T | C | 5.66E-03 | 770.503 | -0.288 | 0.010 | 1.39E-169 | -0.045 | 0.022 | 0.046 |
| TIBC | rs855791 ^a^ | 22 | 37462936 | 0.44 | TMPRSS6 | A | G | 2.27E-04 | 30.786 | 0.026 | 0.005 | 2.88E-08 | -0.024 | 0.012 | 0.045 |
| TIBC | rs9267862 | 6 | 32201269 | 0.05 | NOTCH4 | T | C | 2.42E-04 | 32.765 | 0.055 | 0.010 | 1.04E-08 | -0.014 | 0.029 | 0.635 |
| TIBC | rs9389269 | 6 | 135427159 | 0.27 | HBS1L | C | T | 3.10E-04 | 42.005 | -0.034 | 0.005 | 9.10E-11 | -0.025 | 0.012 | 0.034 |
| TIBC | rs968155 | 6 | 32380715 | 0.43 | TBC1D22B | C | T | 9.95E-04 | 134.911 | -0.055 | 0.005 | 3.45E-31 | -0.008 | 0.013 | 0.555 |

TIBC, total iron binding capacity; Chr, chromosome; Pos, position based on GRCh37/hg19; EAF, effect allele frequency; EA, effect allele; OA, other allele. ^a^ Newly found SNPs in the original meta-analysis of GWAS that associated with each iron status biomarker. ^b^ SNPs that were not found in the outcome dataset and proxy SNPs were used in the final analysis.

S1 Table. Characteristics of SNPs selected as instrumental variables (IVs) for each iron status biomarkers. F values were calculated with the explained variance (R^2^) and sample size (N). F>10 is suggested for powerful enough IVs.
