# Supplementary material for: The causal effect of iron status on risk of anxiety disorders: A two-sample Mendelian randomization study
Source: PLoS One. 2024 Mar 28;19(3):e0300143. doi: 10.1371/journal.pone.0300143 (PMC10977787; doi:10.1371/journal.pone.0300143)
Supplement: S2 Table — (DOCX) [file pone.0300143.s002.docx]

**S2 Table. Original results of MR PRESSO test.**

|  | Exposure | MR Analysis | Causal Estimate | Sd | T-stat | P-value | MR-PRESSO results Global Test | |
| --- | --- | --- | --- | --- | --- | --- | --- | --- |
|  |  |  |  |  |  |  | RSSobs | P value |
| Iron | beta.exposure | Raw | -0.0815004 | 0.04140016 | -1.968602 | 0.05862296 | 45.53397 | 0.0644 |
|  | beta.exposure | Outlier-corrected | NA | NA | NA | NA |  |  |
| Ferritin | beta.exposure | Raw | -0.1358505 | 0.0575265 | -2.361529 | 0.02170333 | 74.77487 | 0.074 |
|  | beta.exposure | Outlier-corrected | NA | NA | NA | NA |  |  |
| Transferrin Saturation | beta.exposure | Raw | -0.1196381 | 0.04129447 | -2.897194 | 0.00791219 | 29.10579 | 0.3502 |
|  | beta.exposure | Outlier-corrected | NA | NA | NA | NA |  |  |
| TIBC | beta.exposure | Raw | 0.07691923 | 0.04530904 | 1.697657 | 0.09927876 | 65.51453 | 0.0018 |
|  | beta.exposure | Outlier-corrected | NA | NA | NA | NA |  |  |

TIBC, total iron binding capacity

S2 Table. Original output of MR PRESSO test from R project.
