# Supplementary material for: The causal effect of iron status on risk of anxiety disorders: A two-sample Mendelian randomization study
Source: PLoS One. 2024 Mar 28;19(3):e0300143. doi: 10.1371/journal.pone.0300143 (PMC10977787; doi:10.1371/journal.pone.0300143)
Supplement: S3 Table — (DOCX) [file pone.0300143.s003.docx]

**S3 Table. Characteristics of IVs in outcome dataset.**

|  | SNP | Beta | SE | P | EAF.outcome | EA.outcome | OA.outcome | proxy.outcome | target_snp.outcome | proxy_snp.outcome | target_a1.outcome | target_a2.outcome | proxy_a1.outcome | proxy_a2.outcome |
| --- | --- | --- | --- | --- | --- | --- | --- | --- | --- | --- | --- | --- | --- | --- |
| Iron | rs10421599 | 0.0153 | 0.0136 | 0.2604 | 0.2167 | A | G | NA | NA | NA | NA | NA | NA | NA |
|  | rs10822143 | 0.004 | 0.0112 | 0.7228 | 0.484 | T | C | TRUE | rs10822143 | rs10822145 | C | T | C | T |
|  | rs114708114 | -0.0224 | 0.0888 | 0.8009 | 0.00414 | T | C | NA | NA | NA | NA | NA | NA | NA |
|  | rs116009877 | 0.0362 | 0.0303 | 0.2325 | 0.03572 | A | G | NA | NA | NA | NA | NA | NA | NA |
|  | rs116169498 | 0.0015 | 0.0536 | 0.9778 | 0.01149 | G | C | NA | NA | NA | NA | NA | NA | NA |
|  | rs117718169 | 0.0016 | 0.0421 | 0.9691 | 0.01914 | T | C | NA | NA | NA | NA | NA | NA | NA |
|  | rs117753190 | -0.0239 | 0.0555 | 0.666699 | 0.01049 | G | C | NA | NA | NA | NA | NA | NA | NA |
|  | rs12206204 | 0.0208 | 0.0457 | 0.6484 | 0.01583 | T | C | NA | NA | NA | NA | NA | NA | NA |
|  | rs12633819 | -0.0061 | 0.0125 | 0.6244 | 0.2756 | G | A | NA | NA | NA | NA | NA | NA | NA |
|  | rs12718598 | -0.016 | 0.0114 | 0.1623 | 0.3985 | C | T | NA | NA | NA | NA | NA | NA | NA |
|  | rs12730935 | -0.0132 | 0.0123 | 0.2828 | 0.2967 | A | G | TRUE | rs12730935 | rs61812598 | A | G | A | G |
|  | rs12975762 | 0.0224 | 0.0133 | 0.0916305 | 0.2328 | G | A | NA | NA | NA | NA | NA | NA | NA |
|  | rs13007705 | 0.0119 | 0.0115 | 0.3019 | 0.3841 | T | C | NA | NA | NA | NA | NA | NA | NA |
|  | rs140393761 | -0.0157 | 0.0534 | 0.7696 | 0.01144 | G | A | NA | NA | NA | NA | NA | NA | NA |
|  | rs145496147 | -0.1135 | 0.0394 | 0.00398199 | 0.02085 | T | C | NA | NA | NA | NA | NA | NA | NA |
|  | rs1799945 | -0.0218 | 0.018 | 0.2253 | 0.1099 | G | C | NA | NA | NA | NA | NA | NA | NA |
|  | rs1800562 | 0.0288 | 0.0295 | 0.3283 | 0.03705 | A | G | NA | NA | NA | NA | NA | NA | NA |
|  | rs1958078 | 0.0184 | 0.0208 | 0.376 | 0.9191 | C | A | NA | NA | NA | NA | NA | NA | NA |
|  | rs35945185 | 0.0245 | 0.0112 | 0.0293002 | 0.4669 | A | G | TRUE | rs35945185 | rs11208711 | A | G | T | G |
|  | rs4774514 | 0.0271 | 0.029 | 0.3509 | 0.03918 | T | C | NA | NA | NA | NA | NA | NA | NA |
|  | rs4854760 | 0.0183 | 0.0123 | 0.136 | 0.295 | G | A | NA | NA | NA | NA | NA | NA | NA |
|  | rs563027675 | 0.0222 | 0.0331 | 0.5021 | 0.03051 | G | C | NA | NA | NA | NA | NA | NA | NA |
|  | rs56912861 | -0.006 | 0.0113 | 0.592 | 0.4689 | A | G | NA | NA | NA | NA | NA | NA | NA |
|  | rs7385804 | -0.0022 | 0.0113 | 0.843 | 0.5525 | A | C | NA | NA | NA | NA | NA | NA | NA |
|  | rs7837764 | 0.019 | 0.0116 | 0.1011 | 0.38 | C | G | NA | NA | NA | NA | NA | NA | NA |
|  | rs855791 | -0.0235 | 0.0117 | 0.0450298 | 0.6526 | G | A | NA | NA | NA | NA | NA | NA | NA |
|  | rs9265878 | -0.0044 | 0.0128 | 0.731799 | 0.2782 | A | G | NA | NA | NA | NA | NA | NA | NA |
|  | rs9295767 | 0.0072 | 0.032 | 0.8223 | 0.0317 | A | G | NA | NA | NA | NA | NA | NA | NA |
|  | rs9399136 | -0.0266 | 0.0118 | 0.0246201 | 0.3405 | C | T | TRUE | rs9399136 | rs34164109 | C | T | T | C |
|  | rs9610638 | -0.0248 | 0.0114 | 0.0286702 | 0.5742 | C | T | TRUE | rs9610638 | rs2072860 | T | C | G | A |
| Ferritin | rs10263500 | -0.0037 | 0.0112 | 0.7411 | 0.4984 | T | C | NA | NA | NA | NA | NA | NA | NA |
|  | rs10750215 | 0.016 | 0.0115 | 0.1649 | 0.3936 | T | G | NA | NA | NA | NA | NA | NA | NA |
|  | rs10801913 | 0.0097 | 0.0122 | 0.4305 | 0.6982 | G | A | NA | NA | NA | NA | NA | NA | NA |
|  | rs10804630 | 0.0024 | 0.0161 | 0.8797 | 0.1436 | C | T | NA | NA | NA | NA | NA | NA | NA |
|  | rs11634990 | 0.0046 | 0.015 | 0.758 | 0.1679 | C | T | NA | NA | NA | NA | NA | NA | NA |
|  | rs12419620 | 0.0011 | 0.0161 | 0.9455 | 0.1409 | G | T | NA | NA | NA | NA | NA | NA | NA |
|  | rs1250244 | -0.0112 | 0.014 | 0.4244 | 0.7979 | C | G | NA | NA | NA | NA | NA | NA | NA |
|  | rs12568930 | 0.0061 | 0.0197 | 0.757 | 0.09023 | C | T | NA | NA | NA | NA | NA | NA | NA |
|  | rs12916360 | 0.0057 | 0.0133 | 0.6674 | 0.2295 | T | G | NA | NA | NA | NA | NA | NA | NA |
|  | rs13253974 | 0.0213 | 0.0124 | 0.08555 | 0.2938 | A | G | NA | NA | NA | NA | NA | NA | NA |
|  | rs143922167 | 0.0648 | 0.0593 | 0.2751 | 0.009354 | A | G | NA | NA | NA | NA | NA | NA | NA |
|  | rs144861591 | 0.0341 | 0.0292 | 0.2422 | 0.03796 | T | C | NA | NA | NA | NA | NA | NA | NA |
|  | rs161044 | 0.0465 | 0.0372 | 0.2112 | 0.9761 | C | T | NA | NA | NA | NA | NA | NA | NA |
|  | rs1694067 | 0.0253 | 0.0116 | 0.0288503 | 0.6267 | C | T | TRUE | rs1694067 | rs895356 | T | C | G | A |
|  | rs17050272 | -0.0243 | 0.0113 | 0.0311903 | 0.5352 | A | G | NA | NA | NA | NA | NA | NA | NA |
|  | rs17112021 | 0.0135 | 0.014 | 0.3342 | 0.2021 | G | T | NA | NA | NA | NA | NA | NA | NA |
|  | rs17476364 | -0.0161 | 0.0239 | 0.4998 | 0.05909 | C | T | NA | NA | NA | NA | NA | NA | NA |
|  | rs17676097 | -0.0096 | 0.012 | 0.4257 | 0.3198 | G | A | NA | NA | NA | NA | NA | NA | NA |
|  | rs1799945 | -0.0218 | 0.018 | 0.2253 | 0.1099 | G | C | NA | NA | NA | NA | NA | NA | NA |
|  | rs1800562 | 0.0288 | 0.0295 | 0.3283 | 0.03705 | A | G | NA | NA | NA | NA | NA | NA | NA |
|  | rs1894692 | 0.0738 | 0.0396 | 0.0626902 | 0.9798 | A | G | NA | NA | NA | NA | NA | NA | NA |
|  | rs199138 | -0.0412 | 0.0219 | 0.0603601 | 0.9299 | G | A | NA | NA | NA | NA | NA | NA | NA |
|  | rs2008598 | 1.00E-04 | 0.0146 | 0.9951 | 0.8184 | A | G | NA | NA | NA | NA | NA | NA | NA |
|  | rs2529440 | 0.0076 | 0.0114 | 0.5023 | 0.4039 | T | C | NA | NA | NA | NA | NA | NA | NA |
|  | rs2595582 | 0.0157 | 0.0119 | 0.1877 | 0.3304 | A | G | NA | NA | NA | NA | NA | NA | NA |
|  | rs28715334 | 0.0079 | 0.0156 | 0.6138 | 0.1536 | T | G | NA | NA | NA | NA | NA | NA | NA |
|  | rs34523089 | -0.0494 | 0.0154 | 0.001309 | 0.1587 | T | C | NA | NA | NA | NA | NA | NA | NA |
|  | rs35107257 | -0.0029 | 0.0298 | 0.9213 | 0.03677 | A | G | NA | NA | NA | NA | NA | NA | NA |
|  | rs36184164 | 0.012 | 0.0198 | 0.545301 | 0.09065 | G | T | NA | NA | NA | NA | NA | NA | NA |
|  | rs370631 | -0.0238 | 0.0145 | 0.1009 | 0.1992 | C | T | NA | NA | NA | NA | NA | NA | NA |
|  | rs45520632 | -0.0424 | 0.0385 | 0.2707 | 0.02224 | C | T | NA | NA | NA | NA | NA | NA | NA |
|  | rs4789111 | -0.0184 | 0.0175 | 0.2938 | 0.8819 | C | T | NA | NA | NA | NA | NA | NA | NA |
|  | rs4808802 | -0.0302 | 0.0135 | 0.0257401 | 0.218 | C | G | NA | NA | NA | NA | NA | NA | NA |
|  | rs4841429 | 0.0156 | 0.0202 | 0.4385 | 0.08804 | G | A | NA | NA | NA | NA | NA | NA | NA |
|  | rs4938939 | -0.0088 | 0.0134 | 0.5124 | 0.2278 | A | G | NA | NA | NA | NA | NA | NA | NA |
|  | rs532436 | 0.0158 | 0.0139 | 0.2553 | 0.2009 | A | G | NA | NA | NA | NA | NA | NA | NA |
|  | rs55778511 | -0.0036 | 0.0217 | 0.868 | 0.07365 | G | T | NA | NA | NA | NA | NA | NA | NA |
|  | rs56206139 | -0.0178 | 0.0312 | 0.568 | 0.03377 | C | A | NA | NA | NA | NA | NA | NA | NA |
|  | rs590097 | -0.006 | 0.0116 | 0.6029 | 0.6155 | G | T | NA | NA | NA | NA | NA | NA | NA |
|  | rs6059696 | 0.0072 | 0.0139 | 0.6031 | 0.205 | G | C | NA | NA | NA | NA | NA | NA | NA |
|  | rs62074125 | -0.0109 | 0.0131 | 0.4084 | 0.2434 | C | A | NA | NA | NA | NA | NA | NA | NA |
|  | rs6760824 | -0.0208 | 0.013 | 0.1106 | 0.7543 | C | A | NA | NA | NA | NA | NA | NA | NA |
|  | rs6822746 | -0.0012 | 0.0115 | 0.9141 | 0.3827 | A | G | NA | NA | NA | NA | NA | NA | NA |
|  | rs7068127 | 0.012 | 0.0116 | 0.2972 | 0.3766 | G | A | NA | NA | NA | NA | NA | NA | NA |
|  | rs708686 | -2.00E-04 | 0.0119 | 0.9861 | 0.334 | T | C | NA | NA | NA | NA | NA | NA | NA |
|  | rs71537957 | -0.0279 | 0.0119 | 0.0188499 | 0.3338 | T | C | NA | NA | NA | NA | NA | NA | NA |
|  | rs72606621 | -0.0091 | 0.0119 | 0.4403 | 0.337 | A | G | NA | NA | NA | NA | NA | NA | NA |
|  | rs735831 | -0.0229 | 0.0321 | 0.4761 | 0.03164 | G | T | NA | NA | NA | NA | NA | NA | NA |
|  | rs7596205 | -0.0144 | 0.0191 | 0.4512 | 0.09715 | A | G | NA | NA | NA | NA | NA | NA | NA |
|  | rs75965181 | -0.017 | 0.0507 | 0.736701 | 0.01264 | A | T | NA | NA | NA | NA | NA | NA | NA |
|  | rs7865362 | -0.0154 | 0.012 | 0.1992 | 0.3293 | T | C | NA | NA | NA | NA | NA | NA | NA |
|  | rs855791 | -0.0235 | 0.0117 | 0.0450298 | 0.6526 | G | A | NA | NA | NA | NA | NA | NA | NA |
|  | rs859788 | 0.0115 | 0.0112 | 0.3042 | 0.4734 | G | A | NA | NA | NA | NA | NA | NA | NA |
|  | rs9512463 | -0.0012 | 0.0129 | 0.9276 | 0.7408 | C | T | NA | NA | NA | NA | NA | NA | NA |
|  | rs970079 | -0.0177 | 0.0119 | 0.1362 | 0.3437 | G | A | NA | NA | NA | NA | NA | NA | NA |
|  | rs9921222 | -0.001 | 0.0112 | 0.928 | 0.4781 | T | C | NA | NA | NA | NA | NA | NA | NA |
|  | rs996347 | -0.0057 | 0.0118 | 0.6285 | 0.3453 | C | T | NA | NA | NA | NA | NA | NA | NA |
| Transferrin Saturation | rs10421599 | 0.0153 | 0.0136 | 0.2604 | 0.2167 | A | G | NA | NA | NA | NA | NA | NA | NA |
|  | rs114708114 | -0.0224 | 0.0888 | 0.8009 | 0.00414 | T | C | NA | NA | NA | NA | NA | NA | NA |
|  | rs115314215 | 0.0256 | 0.0315 | 0.4162 | 0.03337 | T | C | NA | NA | NA | NA | NA | NA | NA |
|  | rs116272812 | -0.0177 | 0.0167 | 0.2905 | 0.1285 | C | T | NA | NA | NA | NA | NA | NA | NA |
|  | rs117718169 | 0.0016 | 0.0421 | 0.9691 | 0.01914 | T | C | NA | NA | NA | NA | NA | NA | NA |
|  | rs12478088 | 0.007 | 0.0114 | 0.5391 | 0.5702 | A | G | NA | NA | NA | NA | NA | NA | NA |
|  | rs12633819 | -0.0061 | 0.0125 | 0.6244 | 0.2756 | G | A | NA | NA | NA | NA | NA | NA | NA |
|  | rs12975762 | 0.0224 | 0.0133 | 0.0916305 | 0.2328 | G | A | NA | NA | NA | NA | NA | NA | NA |
|  | rs13007705 | 0.0119 | 0.0115 | 0.3019 | 0.3841 | T | C | NA | NA | NA | NA | NA | NA | NA |
|  | rs1799945 | -0.0218 | 0.018 | 0.2253 | 0.1099 | G | C | NA | NA | NA | NA | NA | NA | NA |
|  | rs1800562 | 0.0288 | 0.0295 | 0.3283 | 0.03705 | A | G | NA | NA | NA | NA | NA | NA | NA |
|  | rs28487964 | 0.0865 | 0.0449 | 0.05423 | 0.0159 | A | T | NA | NA | NA | NA | NA | NA | NA |
|  | rs35945185 | 0.0245 | 0.0112 | 0.0293002 | 0.4669 | A | G | TRUE | rs35945185 | rs11208711 | A | G | T | G |
|  | rs4774514 | 0.0271 | 0.029 | 0.3509 | 0.03918 | T | C | NA | NA | NA | NA | NA | NA | NA |
|  | rs56912861 | -0.006 | 0.0113 | 0.592 | 0.4689 | A | G | NA | NA | NA | NA | NA | NA | NA |
|  | rs62396224 | -0.0087 | 0.0226 | 0.6997 | 0.06655 | G | A | NA | NA | NA | NA | NA | NA | NA |
|  | rs6456691 | 3.00E-04 | 0.0166 | 0.9839 | 0.8661 | T | C | NA | NA | NA | NA | NA | NA | NA |
|  | rs6592965 | -0.0104 | 0.012 | 0.3894 | 0.3207 | A | G | NA | NA | NA | NA | NA | NA | NA |
|  | rs72838865 | -0.0362 | 0.0436 | 0.406 | 0.0176 | A | C | NA | NA | NA | NA | NA | NA | NA |
|  | rs7385804 | -0.0022 | 0.0113 | 0.843 | 0.5525 | A | C | NA | NA | NA | NA | NA | NA | NA |
|  | rs74338506 | -0.0396 | 0.0378 | 0.2937 | 0.02229 | A | G | NA | NA | NA | NA | NA | NA | NA |
|  | rs7648210 | 0.0186 | 0.0131 | 0.1563 | 0.7552 | G | A | NA | NA | NA | NA | NA | NA | NA |
|  | rs7650925 | -0.0098 | 0.0112 | 0.3829 | 0.4813 | T | G | TRUE | rs7650925 | rs6439441 | G | T | A | G |
|  | rs7837764 | 0.019 | 0.0116 | 0.1011 | 0.38 | C | G | NA | NA | NA | NA | NA | NA | NA |
|  | rs8177271 | 0.0164 | 0.0121 | 0.1777 | 0.3044 | A | G | NA | NA | NA | NA | NA | NA | NA |
|  | rs855791 | -0.0235 | 0.0117 | 0.0450298 | 0.6526 | G | A | NA | NA | NA | NA | NA | NA | NA |
|  | rs9399136 | -0.0266 | 0.0118 | 0.0246201 | 0.3405 | C | T | TRUE | rs9399136 | rs34164109 | C | T | T | C |
|  | rs9610638 | -0.0248 | 0.0114 | 0.0286702 | 0.5742 | C | T | TRUE | rs9610638 | rs2072860 | T | C | G | A |
| TIBC | rs1106735 | 0.0382 | 0.0156 | 0.0143599 | 0.1528 | G | A | NA | NA | NA | NA | NA | NA | NA |
|  | rs112466891 | 0.0123 | 0.0126 | 0.33 | 0.7257 | A | C | TRUE | rs112466891 | rs11709321 | C | A | T | G |
|  | rs112727702 | -0.0205 | 0.0136 | 0.1324 | 0.2153 | T | G | TRUE | rs112727702 | rs2288921 | T | G | G | T |
|  | rs116272812 | -0.0177 | 0.0167 | 0.2905 | 0.1285 | C | T | NA | NA | NA | NA | NA | NA | NA |
|  | rs12206077 | -0.0092 | 0.0126 | 0.4626 | 0.2803 | A | G | NA | NA | NA | NA | NA | NA | NA |
|  | rs12976652 | -0.0125 | 0.0229 | 0.5844 | 0.06384 | C | T | NA | NA | NA | NA | NA | NA | NA |
|  | rs13008704 | 0.0165 | 0.0112 | 0.1407 | 0.4861 | T | C | NA | NA | NA | NA | NA | NA | NA |
|  | rs13084306 | 0.0309 | 0.0506 | 0.5408 | 0.01251 | A | C | NA | NA | NA | NA | NA | NA | NA |
|  | rs143530446 | -0.0019 | 0.0598 | 0.9742 | 0.00915 | T | C | NA | NA | NA | NA | NA | NA | NA |
|  | rs174547 | 0.0327 | 0.0114 | 0.00406303 | 0.4138 | C | T | NA | NA | NA | NA | NA | NA | NA |
|  | rs17580 | -0.1126 | 0.0592 | 0.0574394 | 0.009198 | A | T | NA | NA | NA | NA | NA | NA | NA |
|  | rs17767742 | 0.0047 | 0.0121 | 0.7009 | 0.3097 | G | C | NA | NA | NA | NA | NA | NA | NA |
|  | rs1799945 | -0.0218 | 0.018 | 0.2253 | 0.1099 | G | C | NA | NA | NA | NA | NA | NA | NA |
|  | rs1800562 | 0.0288 | 0.0295 | 0.3283 | 0.03705 | A | G | NA | NA | NA | NA | NA | NA | NA |
|  | rs1927693 | -0.0195 | 0.0117 | 0.0963008 | 0.3691 | A | G | NA | NA | NA | NA | NA | NA | NA |
|  | rs199138 | -0.0412 | 0.0219 | 0.0603601 | 0.9299 | G | A | NA | NA | NA | NA | NA | NA | NA |
|  | rs2236252 | 0.003 | 0.0158 | 0.8479 | 0.1473 | T | C | NA | NA | NA | NA | NA | NA | NA |
|  | rs35570672 | 0.0062 | 0.0129 | 0.6303 | 0.7498 | C | T | NA | NA | NA | NA | NA | NA | NA |
|  | rs35769520 | 0.0251 | 0.0116 | 0.0305703 | 0.3645 | A | G | NA | NA | NA | NA | NA | NA | NA |
|  | rs469882 | 0.0089 | 0.0137 | 0.515 | 0.2126 | C | A | NA | NA | NA | NA | NA | NA | NA |
|  | rs4846335 | -0.0173 | 0.016 | 0.2796 | 0.1444 | A | C | NA | NA | NA | NA | NA | NA | NA |
|  | rs56195124 | -0.0395 | 0.0325 | 0.2234 | 0.03249 | A | G | NA | NA | NA | NA | NA | NA | NA |
|  | rs59950280 | 0.0086 | 0.0132 | 0.513499 | 0.2448 | A | G | NA | NA | NA | NA | NA | NA | NA |
|  | rs6025 | 0.0761 | 0.0397 | 0.0550098 | 0.97974 | C | T | NA | NA | NA | NA | NA | NA | NA |
|  | rs62183592 | -0.0347 | 0.0297 | 0.2436 | 0.03879 | T | C | NA | NA | NA | NA | NA | NA | NA |
|  | rs72840508 | -0.0301 | 0.0429 | 0.484 | 0.01799 | T | A | NA | NA | NA | NA | NA | NA | NA |
|  | rs7297861 | 0.008 | 0.0171 | 0.641899 | 0.1227 | C | T | NA | NA | NA | NA | NA | NA | NA |
|  | rs7432894 | 0.0247 | 0.0127 | 0.0510305 | 0.7277 | T | C | NA | NA | NA | NA | NA | NA | NA |
|  | rs8177257 | -0.0447 | 0.0224 | 0.0463895 | 0.06676 | T | C | NA | NA | NA | NA | NA | NA | NA |
|  | rs855791 | -0.0235 | 0.0117 | 0.0450298 | 0.6526 | G | A | NA | NA | NA | NA | NA | NA | NA |
|  | rs9267862 | -0.0138 | 0.0291 | 0.6354 | 0.03902 | T | C | TRUE | rs9267862 | rs9267881 | T | C | A | C |
|  | rs9389269 | -0.025 | 0.0118 | 0.0340902 | 0.3428 | C | T | NA | NA | NA | NA | NA | NA | NA |
|  | rs968155 | -0.0077 | 0.013 | 0.554501 | 0.5598 | T | C | NA | NA | NA | NA | NA | NA | NA |

TIBC, total iron binding capacity; EAF, effect allele frequency; EA, effect allele; OA, other allele.

S3 Table. Characteristics of IVs in outcome dataset.
