# Supplementary figures and images for: The causal effect of iron status on risk of anxiety disorders: A two-sample Mendelian randomization study
Source: PLoS One. 2024 Mar 28;19(3):e0300143. doi: 10.1371/journal.pone.0300143 (PMC10977787; doi:10.1371/journal.pone.0300143)

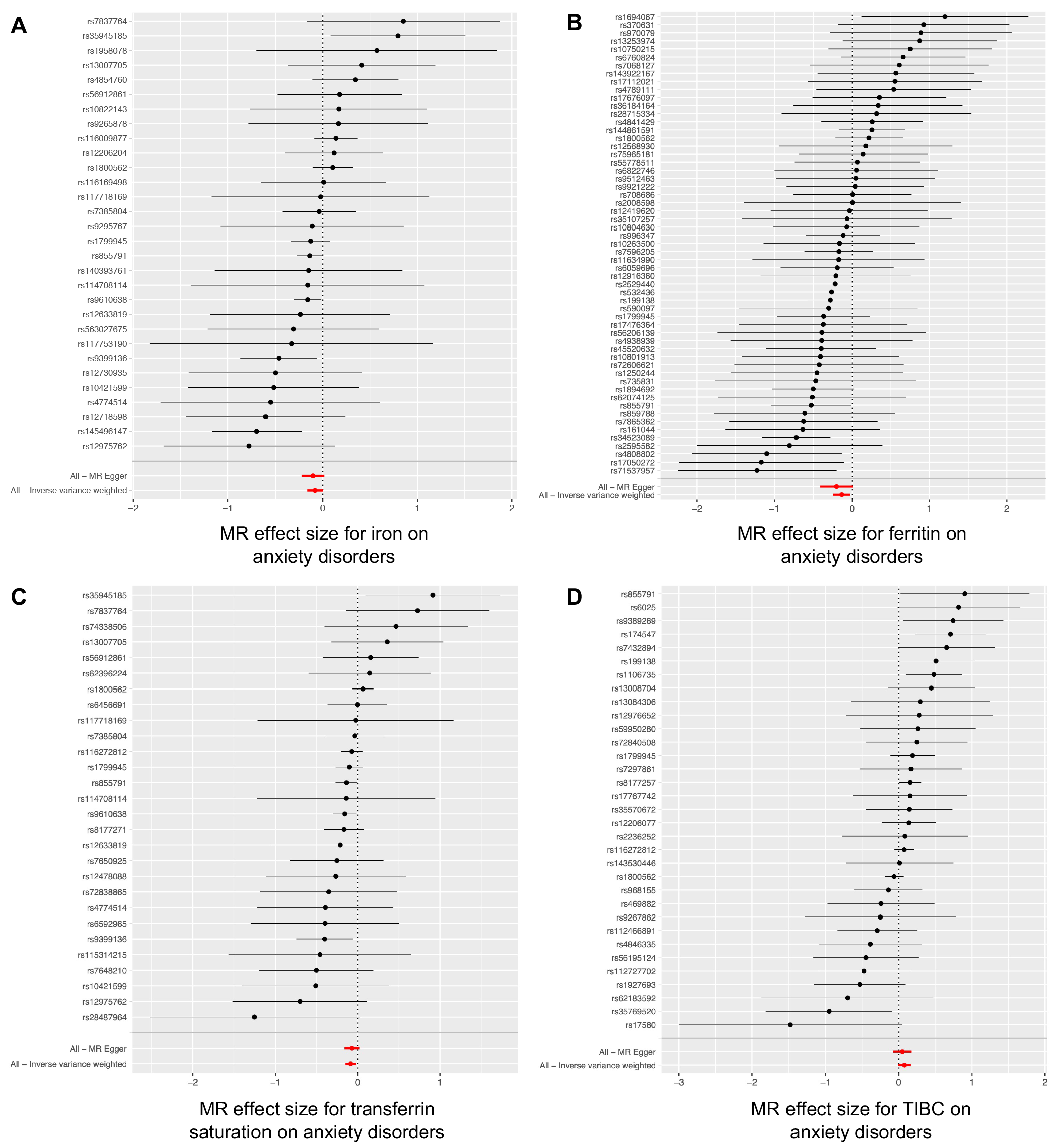

Supplement: S1 Fig — Forest plots for the effects of serum iron (A), ferritin (B), transferrin (C) and TIBC (D) associated SNPs on anxiety disorders. (TIF) [file pone.0300143.s004.tif]

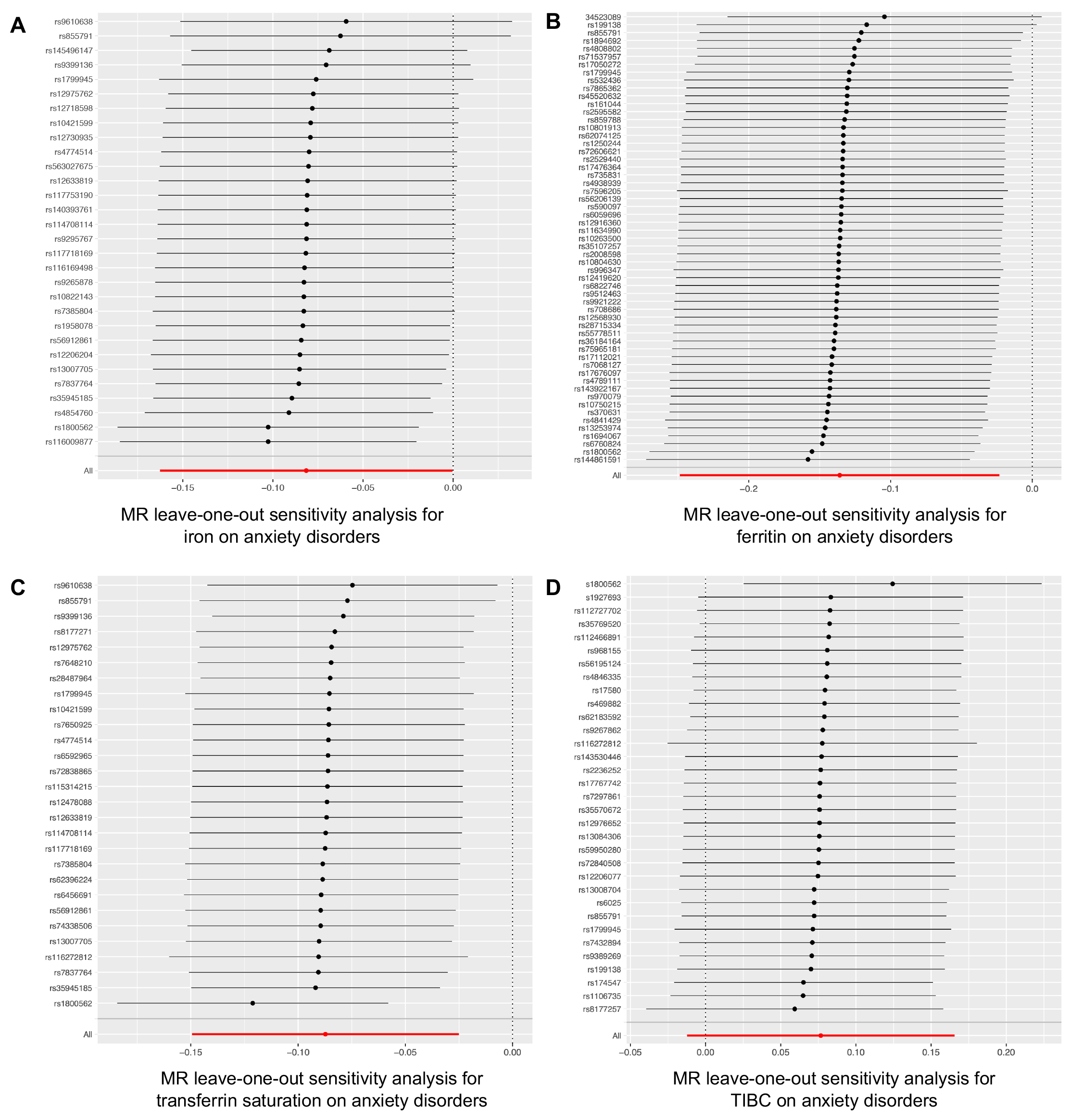

Supplement: S2 Fig — Leave-one-out plots for the effects of serum iron (A), ferritin (B), transferrin (C) and TIBC (D) associated SNPs on anxiety disorders. (TIF) [file pone.0300143.s005.tif]

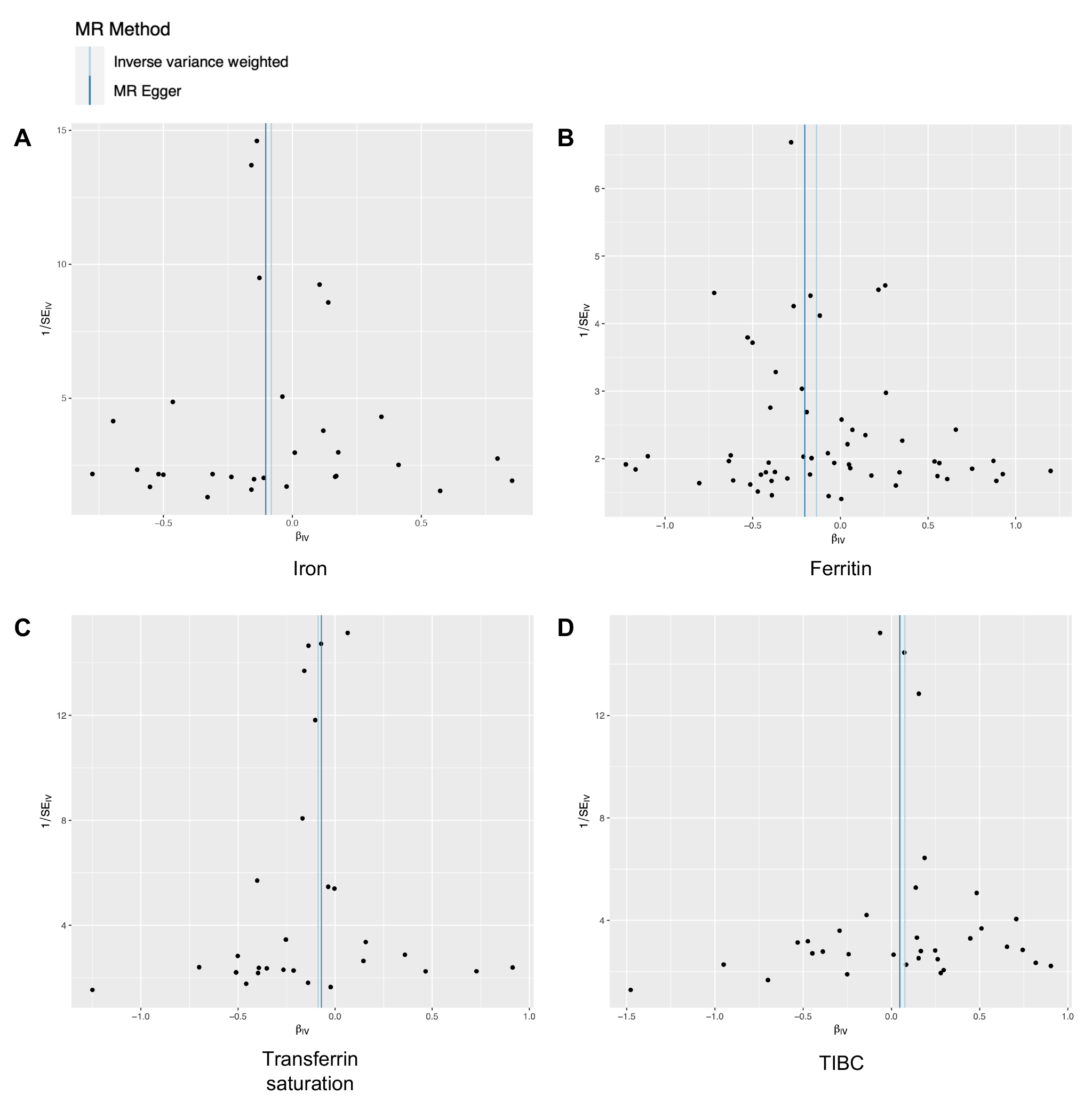

Supplement: S3 Fig — Funnel plots for the effects of serum iron (A), ferritin (B), transferrin (C) and TIBC (D) associated SNPs on anxiety disorders. (TIF) [file pone.0300143.s006.tif]
