# Supplementary material for: The causal effect of iron status on risk of anxiety disorders: A two-sample Mendelian randomization study
Source: PLoS One. 2024 Mar 28;19(3):e0300143. doi: 10.1371/journal.pone.0300143 (PMC10977787; doi:10.1371/journal.pone.0300143)
Supplement: S1 File — (DOCX) [file pone.0300143.s007.docx]

**R code**

(Take the data of serum iron level for example; statements with underline are the illustration of the subsequent code)

#Read local data

exp_dat_iron <- read_exposure_data(

filename = "Ironhomeostasis_Iron_IasterX_2018-11-07.wInfo.cl.txt",

sep = "\t",

snp_col = "UK10Kid",

beta_col = "Beta",

effect_allele_col = "UK10Kea",

other_allele_col = "UK10Koa",

eaf_col = "UK10Keaf",

pval_col = "P")

#Selection of SNPs with p value < 5e-8

exp_dat_iron <- exp_dat_iron[exp_dat_iron$pval.exposure < 5e-8,]

#Findout SNPs in Linkage Disequilibrium

exp_dat_iron <- clump_data(exp_dat_iron)

#Export data

write.csv(exp_dat_iron, file="exp_dat_iron.csv")

#Calculation of se value for each SNP

exp_dat_iron<-read.csv(file="exp_dat_iron.csv",header=TRUE,sep=",")

beta<-exp_dat_iron$beta.exposure

P<-exp_dat_iron$pval.exposure

se=numeric(length=length(exp_dat_iron$beta.exposure))

for(i in 1:length(exp_dat_iron$beta.exposure)) se[i]=sqrt(((beta[i])^2)/qchisq(P[i],1,lower.tail = F))

se

#Extract outcome data

out_dat_iron <- extract_outcome_data(

snps = exp_dat_iron$SNP,

outcomes = "finn-b-KRA_PSY_ANXIETY")

#Export data

write.csv(out_dat_iron, file="out_dat_iron.csv")

#Harmonise data

dat_irontoAnxiety <- harmonise_data(

exposure_dat = exp_dat_iron,

outcome_dat = out_dat_iron)

#Export data

write.csv(dat_irontoAnxiety, file="dat_irontoAnxiety.csv")

#Findout outliers by MR PRESSO

run_mr_presso(dat_irontoAnxiety, NbDistribution = 5000)

#MR analysis

res_irontoAnxiety <- mr(dat_irontoAnxiety)

res_irontoAnxiety

write.csv(res_irontoAnxiety, file="res_irontoAnxiety.csv")

#IVW method with fixed effect

res_irontoAnxiety_fixed<-mr(dat_irontoAnxiety,method_list=c('mr_ivw_fe'))

#Generating odds ratios

generate_odds_ratios(res_irontoAnxiety)

generate_odds_ratios(res_irontoAnxiety_fixed)

#Heterogeneity test

mr_heterogeneity(dat_irontoAnxiety)

#Pleiotropy test

mr_pleiotropy_test(dat_irontoAnxiety)

#MR analysis on each SNP individually

res_single <- mr_singlesnp(dat_irontoAnxiety)

res_single

#Leave one out sensitivity analysis

res_loo <- mr_leaveoneout(dat_irontoAnxiety)

res_loo

#Generating and saving plot for MR and sensitivity analysis results

p1 <- mr_scatter_plot(res_irontoAnxiety, dat_irontoAnxiety)

p1[[1]]

library(ggplot2)

ggsave(p1[[1]], file="res_irontoAnxiety.pdf", width=7, height=7)

p2 <- mr_forest_plot(res_single)

p2[[1]]

ggsave(p2[[1]], file="forest_plot_irontoAnxiety.pdf", width=7, height=7)

p3 <- mr_leaveoneout_plot(res_loo)

p3[[1]]

ggsave(p3[[1]], file="leaveoneout_plot_irontoAnxiety.pdf", width=7, height=7)

p4 <- mr_funnel_plot(res_single)

p4[[1]]

ggsave(p4[[1]], file="funnel_plot_irontoAnxiety.pdf", width=7, height=7)
